# Myosin Vb Mediated Plasma Membrane Homeostasis Regulates Peridermal Cell Size and Maintains Tissue Homeostasis in the Zebrafish Epidermis

**DOI:** 10.1371/journal.pgen.1004614

**Published:** 2014-09-18

**Authors:** Sonal, Jaydeep Sidhaye, Mandar Phatak, Shamik Banerjee, Aditya Mulay, Ojas Deshpande, Sourabh Bhide, Tressa Jacob, Ines Gehring, Christiane Nuesslein-Volhard, Mahendra Sonawane

**Affiliations:** 1 Department of Biological Sciences, Tata Institute of Fundamental Research, Colaba, Mumbai, India; 2 Indian Institute of Science Education and Research, Pune, India; 3 Department of Genetics, Max-Planck Institute for Developmental Biology, Tuebingen, Germany; Cologne University, Germany

## Abstract

The epidermis is a stratified epithelium, which forms a barrier to maintain the internal milieu in metazoans. Being the outermost tissue, growth of the epidermis has to be strictly coordinated with the growth of the embryo. The key parameters that determine tissue growth are cell number and cell size. So far, it has remained unclear how the size of epidermal cells is maintained and whether it contributes towards epidermal homeostasis. We have used genetic analysis in combination with cellular imaging to show that zebrafish *goosepimples/myosin Vb* regulates plasma membrane homeostasis and is involved in maintenance of cell size in the periderm, the outermost epidermal layer. The decrease in peridermal cell size in Myosin Vb deficient embryos is compensated by an increase in cell number whereas decrease in cell number results in the expansion of peridermal cells, which requires *myosin Vb (myoVb)* function. Inhibition of cell proliferation as well as cell size expansion results in increased lethality in larval stages suggesting that this two-way compensatory mechanism is essential for growing larvae. Our analyses unravel the importance of Myosin Vb dependent cell size regulation in epidermal homeostasis and demonstrate that the epidermis has the ability to maintain a dynamic balance between cell size and cell number.

## Author Summary

The epidermis is the outermost epithelial component of the vertebrate skin. It functions as an effective barrier against pathogens and prevents loss of body fluids to the surrounding environment. The factors involved in the maintenance of epidermal architecture have been under intense investigation since the last two decades. Here we report that zebrafish Myosin Vb, a molecular motor, which transports various cargoes inside epithelial cells, is involved in the maintenance of cell size in the outermost epidermal layer called periderm. We show that in the absence of *myosin Vb* function there is perturbed membrane transport and an increase in degradation of membrane components leading to cell shrinkage in the *myosin Vb* mutant. The epidermis compensates for this decrease in cell size, which may compromise epidermal integrity, by increasing the cell number. We also show that in the absence of cell proliferation, the cell size increases to compensate for the decrease in cell number. Simultaneous reduction in cell proliferation as well as cell size results in death of the embryos. Thus, our analyses unravel previously unknown compensatory mechanisms that exist in the epidermis to maintain the tissue integrity.

## Introduction

The epidermis, the outer-most stratified epithelium in metazoans, performs essential functions such as maintenance of body fluids and protection against pathogenic invasion. The epidermis develops from mono-layered non-neural ectoderm during embryogenesis. Initially, it is a bi-layered tissue consisting of the inner basal epidermis and the outer periderm. In mammals, the periderm develops from the basal cells which migrate outwards during early development [1], [2]. In zebrafish the outermost embryonic epithelium, called the enveloping layer, gives rise to the periderm [3]. Tight junctions are an integral part of peridermal cells and contribute to the barrier function [1], [4], [5]. Thus, this early bi-layered epidermis may help in maintaining the interior milieu of the growing vertebrate embryos. The epidermis remains bi-layered during embryonic development in most vertebrates studied. It becomes multilayered before birth in amniotes, including humans, and during metamorphosis in fishes and frog [6], [7], [8], [9], [10].

Being the outermost cover, growth of the epidermis must coordinate with the changes in size and shape of the growing embryo. The tissue growth is achieved either by increase in cell number or cell size or both. The importance of cell number in epidermis development is underscored by the studies done in p63 knockout mice and zebrafish p63 deficient larvae. The loss of p63 function, which is essential for the maintenance of stem cells in stratified epithelia, results in paucity of epidermal cells leading to thinner epidermis in mice and loss of tissue integrity in zebrafish [11], [12], [13], [14], [15], [16]. So far, there is no report on how cell size is maintained in the epidermis, nor how cell size contributes to the maintenance of epidermal homeostasis during development.

Membrane transport is intimately linked with cell size maintenance. It has been shown that endocytosis and exocytosis play crucial roles in regulating the cell surface area [17], [18], [19], [20]. Myosin Vb- an actin based molecular motor- acts as an effector for Rab GTPases Rab8a, Rab10 and Rab11a [21], [22], [23], [24], [25] to regulate exocytosis and recycling of membrane components as well as receptors [23], [26], [27], [28], [29], [30], [31], [32], [33], [34], [35]. Furthermore, Myosin Vb along with Rab10 is involved in membrane biogenesis during axon formation [36]. Recent reports suggest that in neurons, the *myosin V* paralogs act redundantly to regulate neuronal size by controlling transport of PTEN to the plasma membrane [37]. It is not clear whether any of the *myosin V* paralogs regulates cell size in the epidermis.

The functions of Myosin V at the organismal level have been unravelled by performing loss of function studies. In Drosophila, *didum/myosin V* has been implicated in apical transport of Rhodopsin in photoreceptor cells, secretion of proteins 2A12 as well as Pio in trachea and localisation of *oskar* mRNA in the oocyte [38], [39], [40], [41]. Of the three vertebrate paralogs, MyoVa, in association with Rab27a and Melanophilin/Slac2-a, is involved in melanosome trafficking [42], [43], [44], [45], [46], [47]. Consistently, mutations in *myoVa* result in hypo-pigmentation of hair and skin in mammals [45], [48], [49], [50]. In humans this hypo-pigmentation phenotype may exist along with immunological defects or neurological dysfunction and is called Griscelli syndrome [51]. In addition, mutations in *myoVb* – the second *myosin* paralog - in humans are associated with Microvillus Inclusion Disease, which is characterised by inclusion bodies as well as loss of cell polarity in intestinal epithelium resulting in diarrhoea and excessive loss of body fluids [52]. Furthermore, Rab10-Myosin Vb dependent membrane biogenesis plays an essential role in development of optic axonal tracks in zebrafish [36].

We report here that the zebrafish larval epidermis -a simple bi-layered tissue consisting of a basal epidermis and an outer periderm - requires *myoVb* function for maintenance of membrane homeostasis during embryonic development. In *goosepimples/myoVb* deficient embryos, perturbation in plasma membrane homeostasis results in a reduced surface area and smaller size of the peridermal cells. However, this does not compromise the integrity as the loss in cell surface area is compensated by an increase in the number of peridermal cells. In contrast, decrease in cell number by p63 knockdown or by treatment with inhibitors of cell proliferation is compensated by an expansion of the epidermal cell size, which requires *myoVb* function. Notably, reduction of endocytosis in *myoVb* deficient embryos results in restoration of the peridermal cells size and proliferation to the wild type levels. Our analyses reveal an important function for *myosin Vb* in the maintenance of epidermal architecture and unravel a two-way compensatory mechanism in the vertebrate epidermis.

## Results

### The *goosepimples* (*gsp*) locus encodes the actin based molecular motor Myosin Vb

**Figure 1:**
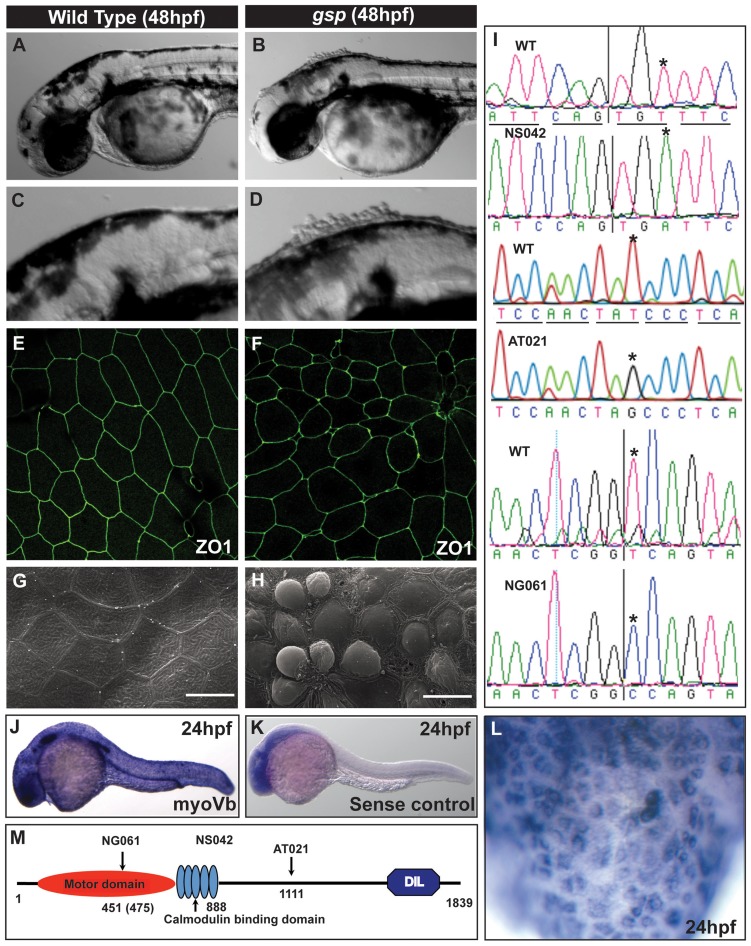
The *gsp* locus encodes for molecular motor Myosin Vb. Representative images of 48hpf wild-type (A,C) and *gsp* mutant larvae (B,D). ZO1 immuno-localisation indicates that as compared to wild type (E) cell shapes are irregular and peridermal cells are smaller in the *gsp* mutant (F). SEM images of wild-type (G) and *gsp* mutant (H) confirm the rounding-up phenotype of peridermal cells. Sequence chromatograms of *gsp^NS042^*, *gsp^AT021^* and *gsp^NG061^* alleles (I). The asterisks in ‘I’ indicate the base substitutions in the mutant alleles. In situ hybridisation using antisense (J) and sense (K) probes against *myoVb*. High magnification image (L) reveal that *myoVb* is expressed in the head peridermal cells. A schematic (M) of domain structure of Myosin Vb indicating the positions of mutations in the three alleles. Note that in NG061 allele the splice site mutation is at 451^st^ aa but the truncation would occur at 475^th^ aa due to a frame-shift. Scale bars in G, H corresponds to 15 µ.

In a mutagenesis screen, performed to identify genes involved in the maintenance of epidermal integrity (Sonawane and Nuesslein-Volhard, unpublished), we isolated two alleles of the previously identified *goosepimples (gsp)* gene [53]. The phenotype of *gsp*, which is characterised by rounding up of epidermal cells, becomes apparent between 36–48 hours post fertilisation (hpf). It is most prominent on the larval head (Figure 1A–D). While a few of the mutant embryos die by 48 hpf, most larvae recover and show a subtle or no morphological phenotype beyond 72 hpf (Figure S1A,B). Immunostaining for the tight junction component, ZO-1 and the adherens junction component E-cadherin confirmed that several of *gsp* peridermal cells exhibit rounded-up morphology in comparison to polygonal shapes in the wild-type (Figure 1E,F). However, there is no prominent effect on the shape of basal epidermal cells in the mutant larvae (Figure S1C,D). Scanning electron microscopy analysis further revealed that at 48 hpf wild type peridermal cells have a flat apical surface whereas in the mutant periderm, cells bulge out of the tissue (Figure 1G,H).

The *gsp* locus was mapped to a narrow interval of 0.2 cM between two z markers, z11532 and z7809, on chromosome 21. We selected *myoVb* as a candidate gene and sequenced the entire cDNA from the three alleles, two from the recent screen and one from the previous screen. In *gsp^NS042^* and *gsp^AT021^* alleles, we found non-sense mutations leading to premature stop at- aa 888 (cysteine) and 1111 (Tyrosine) positions, respectively (Figure 1I). In *gsp^NG061^* we identified a modified transcript with the 10th exon missing (Figure S1E) which leads to a frame-shift resulting in a premature termination of the protein at the 475^th^ amino acid. Sequencing of the genomic locus identified a mutation in the splice donor site flanking exon 10, which results in deletion of exon 10 (Figure 1I). Myosin Vb consists of an ATPase/motor domain, calmodulin binding domains, coiled-coils and a Dilute domain (Figure 1M). Since all three mutations abrogate large parts of the tail, which is involved in cargo binding, we presume a complete loss of motor function in all three alleles. In addition, in these three mutant alleles conserved Rab10 [36] and Rab11 binding sites (Figure S1F) are absent indicating major effect on the membrane recycling and biogenesis activity of Myosin Vb [23], [36]. We further knocked down *myoVb* function using morpholinos designed against the transcriptional start site and the splice donor site, mutated in the NG061 allele. Both the morpholinos recapitulated the *gsp* phenotype (Figure S1G–J).

The zebrafish genome assembly Zv9 shows 2 copies of *myoVb* in the genomic interval where *gsp* maps. We identified two introns- between exon 10 to 11 and exon 17 to18 - which showed different lengths in the two copies. However, the PCR amplification of these two introns from the genomic DNA isolated from mutant larvae did not yield two different products (Figure S2A). Furthermore, by genomic sequencing of a 361 bp region around the NS042 mutation from 5 *gsp* mutant larvae, we did not obtain a wild type peak (for T) along with the mutant peak (for A), which is expected if there are two copies and only one is mutated at this site (Figure S2B). We conclude that there is only one *myoVb* gene in the genomic interval between z11532 and z7809.

We analysed the expression of three *myosin V* paralogs by in situ hybridisation. This analysis revealed that initial ubiquitous expression of *myoVb* during gastrulation becomes restricted to the epidermis, lateral line primordium, otic placode and pronephros by 24 hpf (Figure 1 J–L). By 48hpf the epidermal expression persists only in the head epidermis (Figure S2C). Imaging of 24 and 48 hour old larvae at higher magnification and sectioning of the 48hpf stained larvae revealed that *myoVb* is mainly expressed in the superficial peridermal cells in the epidermis (Figure 1L; Figure S2C,F). Neither *myoVaa*, which show higher homology with murine *myoVa*, nor *myoVc* is expressed in the epidermis (Figure S2D,E). RT-PCR analysis of early cleavage stages revealed that detectable levels of transcripts for both *myoVaa* and *myoVc*, but not for *myoVb*, are deposited maternally (Figure S2G).

To conclude, *gsp* encodes for the actin based molecular motor Myosin Vb. During early embryogenesis, *myoVb* is expressed ubiquitously including in the EVL-the precursor of periderm. At later stages, the expression is observed in the periderm at 24hpf and persists in the dorsal head peridermal cells at 48hpf.

### Recycling endosomes, late endosomes and lysosomes accumulate in the peridermal cells in the absence of *gsp/myoVb* function

Drosophila Myosin V and Myosin Vb in vertebrates have been shown to be essential for the apical secretion and transport of recycling endosomes to the plasma membrane [21], [22], [23], [26], [27], [29], [38], [39], [54]. We hypothesised that perturbation in any of these functions would lead to an increase in the vesicular content in the cytoplasm of the mutant peridermal cells. Indeed, our electron microscopy analysis revealed vesicular bodies of varying sizes in the cytoplasm of the mutant peridermal cells (Figure S3A–D).

**Figure 2:**
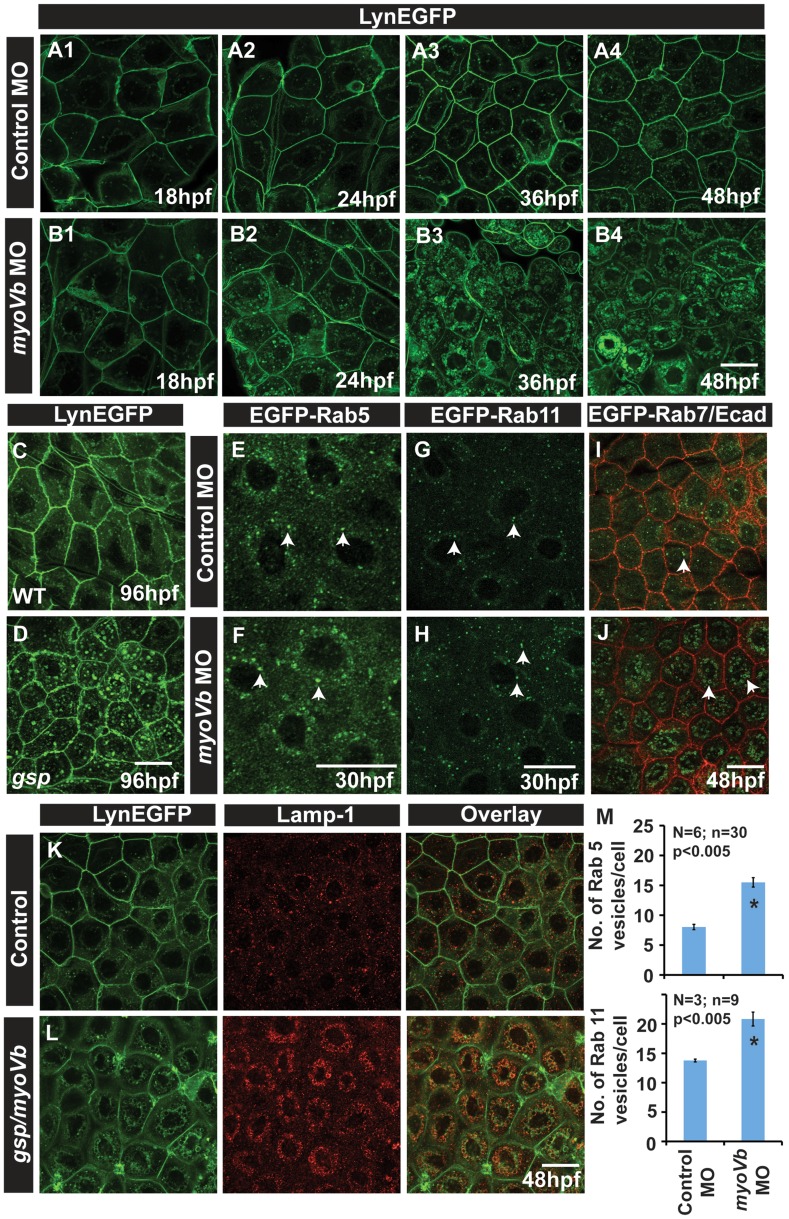
In absence of Myosin Vb, endosomes and lysosomes accumulate in the cytoplasm of peridermal cells. Time course analysis of vesicle accumulation in control (A1–A4) and *myoVb* (B1–B4) morpholino injected embryos using Tg(cldnB:lynEGFP) background. Note that the accumulation of vesicles begins at 24hpf. In comparison to wild-type (WT) larva (C) *gsp* mutant (D) exhibit vesicles in the peridermal cells at 4dpf. Analysis of EGFP-Rab 5 (E,F), EGFP-Rab 11 (G,H), EGFP-Rab7 (I,J) vesicle accumulation at 30 hpf in control morpholino (E,G,I) and *myoVb* morpholino (F,H,J) injected embryos. Note the increase in EGFP-Rab5, EGFP-Rab11 and EGFP-Rab7 labelled endosomes in morphants (F,H,J). Lamp-1 staining in control (K) and *gsp/myoVb* mutant (L) embryos using Tg(cldnB:lynEGFP) background reveals increased lysosomal activity in mutants (L). Quantification of Rab5 and Rab11 vesicles in control and *myoVb* morpholino injected embryos at 30 hpf (M). Arrowheads in E,F indicate EGFP-Rab5 labelled early endosomes; in G,H, EGFP-Rab11 labelled recycling endosomes and in I,J, EGFP-Rab7 labelled late endosomes. Asterisk in ‘M’ indicate that the difference between control and *myoVb* morpholino injected embryos is statistically significant as per student's t test (p≤0.05). Scale bars correspond to 20 µ.

In order to probe the formation of vesicular bodies in a more tractable manner, we used a zebrafish transgenic line in which the *claudin B* promoter drives the expression of lynEGFP in the peridermal cells [55]. Lyn tag is a ten amino acid peptide derived from the Lyn protein belonging to the Src family of kinases. This peptide gets myristylated as well as palmitylated inside the cell and gets tethered into the membranes [56]. The *myoVb* morphants, obtained using splice site morpholino, and *gsp* mutants both exhibited accumulation of vesicular bodies in the peridermal cells labelled with lynEGFP (Figure S3E,F). Since *myoVb* morpholino injected embryos showed a highly specific phenotype indistinguishable from the *gsp* mutant phenotype, we used this morpholino for further analysis. By using morpholino-mediated knock-down of *myoVb* in the Tg(cldnB:lynEGFP) line followed by immunostaining for GFP at different developmental stages, we observed an accumulation of small vesicles in the morphants as early as 24hpf, much before the morphological phenotype becomes apparent (Figure 2A2,B2). The number and size of these vesicular bodies increased over time indicating an increase in the cytoplasmic membranous material at subsequent stages (Figure 2A1–B4). Interestingly, even though the morphological rounding up phenotype subsides, the accumulation of vesicular bodies persists at 4dpf in the *gsp* mutants indicating that the recovery does not happen at the cellular level (Figure 2C,D). Even at 7dpf, vesicles are seen in *gsp* mutants albeit with reduced number as compared to earlier stages (Figure S3G,H).

To further understand the nature of accumulated vesicles, we depleted Myosin Vb levels in transgenic lines, which ubiquitously express EGFP tagged versions of Rab5c, Rab11a and Rab7 [57]. Rab5 and Rab7 mark the early (EE) and late endosome (LE), respectively, whereas Rab11 forms a ternary complex with Myosin Vb and Rab11 family interacting proteins and marks recycling endosome (RE) [21], [22], [58], [59], [60], [61]. While both morphants and control exhibited numerous EEs and REs at 30hpf, our quantification revealed an increase in the number of Rab5 and Rab11 labelled vesicles in the peridermal cells of morphant embryos (Figure 2E–H, M). Although LEs were barely detectable in the wild type peridermal cells, their number and size was substantially increased in the periderm of *myoVb* morphants at 48hpf (Figure 2I,J). To test whether the accumulated endosomes undergo lysosomal degradation, we used Lamp 1, a *bona fide* lysosomal marker, and Lysotracker dye, which exhibits fluorescence in an acidic environment. Stainings revealed that the wild type peridermal cells show rare and isolated spots of Lysotracker/Lamp1 labelled compartments. In comparison, the *myoVb* deficient peridermal cells showed a remarkable increase in lysosomal compartments, accumulated in the perinuclear region (Figure 2K,L; Figure S3I,J). Although not as prominent, this increase in lysosomal activity is present even at 8dpf in the peridermal cell of the *gsp* mutants (Figure S3K,L).

To conclude, the presence of Rab5 and Rab11 endosomes indicates that endocytosis and recycling takes place in the peridermal cells in wild type embryos. However, the lysosomal activity is minimal in wild type peridermal cells presumably due to recycling of endocytosed material or its retrograde transport to Golgi. In the absence of *gsp/myoVb* function, early recycling and late endosomes accumulate in the peridermal cells. The endosomes accumulated in *myoVb* deficient peridermal cells are further targeted for lysosomal degradation. The endosome accumulation precedes the morphological rounding up phenotype and is not a consequence of the loss of peridermal cell shape.

### Endocytosis from apical and basolateral domains of peridermal cells contribute to the endosomal pool in the absence of *myoVb* function

**Figure 3:**
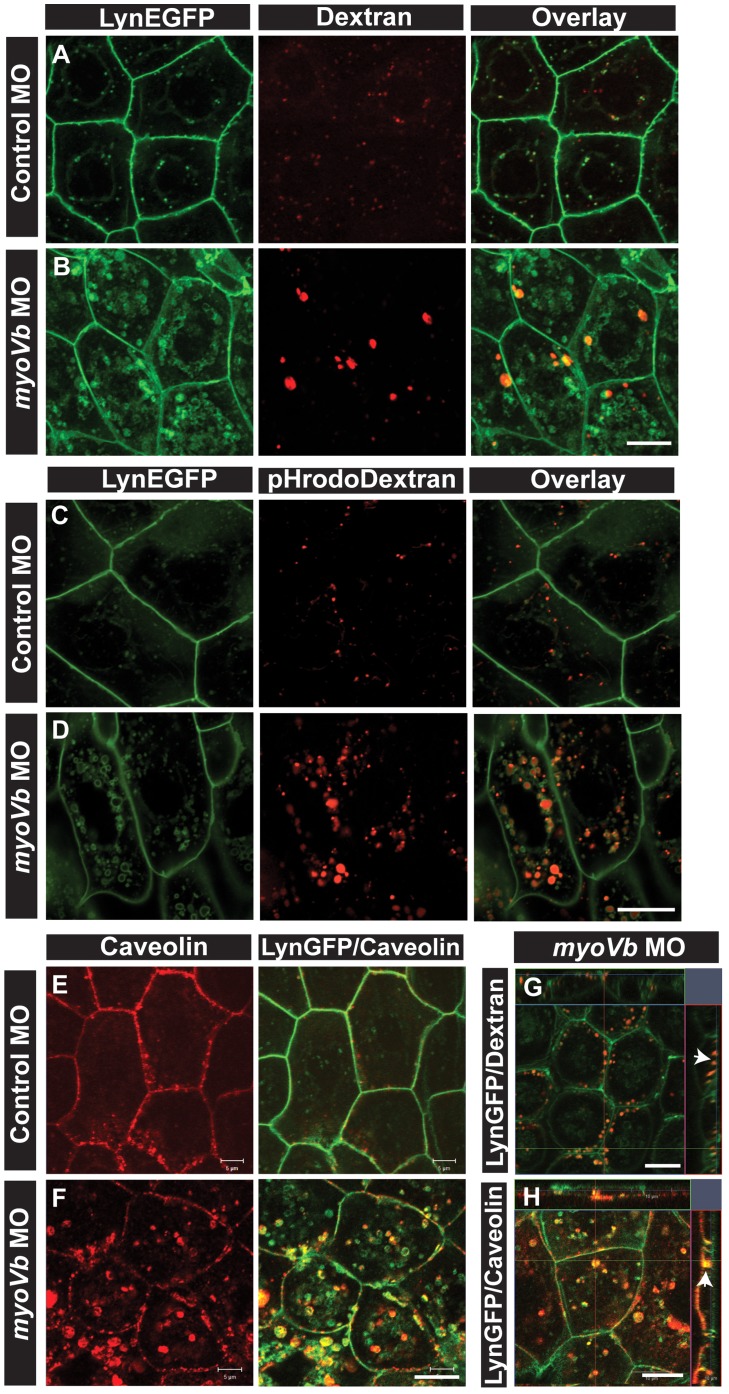
Endocytosis from apical and basolateral domain contributes to endosome and lysosome formation. Uptake of Alexa 546 conjugated Dextran (A,B) and pHrodo Dextran (C,D) by peridermal cells in control (A,C) and *myoVb* (B,D) morpholino injected embryos obtained from Tg(cldnB:lynEGFP) line. Alexa 546 Dextran and pHrodo Dextran accumulates in lynEGFP vesicles in the morphants. Caveolin staining in lynEGFP expressing peridermal cells of control (E) and *myoVb* morphants (F) reveal that endocytosis from basolateral domain contributes for vesicle formation in morphants. X-Y plane and orthogonal projections of Alexa 546 Dextran, lynEGFP (G) and caveolin, lynEGFP (H) labelled morphant peridermal cells. Note the apical localisation of Dextran vesicles (arrowhead in G) and basolateral localization of caveolin vesicle (arrowhead in H). Scale bars are equivalent to 10 µ.

While we demonstrated that the cytoplasm of *gsp/myoVb* deficient peridermal cells is filled with endosomal/lysosomal vesicles, their origin remained unclear. We analysed the uptake and progression of 10 kda Alexa fluor 546-conjugated Dextran in the *myoVb* morphant and control larvae in the Tg(cldnB:lynEGFP) line to ascertain the origin of the endosomes. Incubation of 27hpf larvae for 9 hours revealed small Dextran-positive vesicles in the wild type peridermal cells. In contrast, in *myoVb* morphants Dextran was observed in the large perinuclear vesicles in the peridermal cells (Figure 3A,B). In *myoVb* morphants Dextran filled vesicles were seen enriched at the apical domain but not at the basolateral domain of the peridermal cells (Figure 3G). This observation further suggested that the tracer enters from the apical domain of the peridermal cells. To validate this observation, we followed the uptake of wheat germ agglutinin (WGA) after its binding to the apical surface. WGA binds to surface glycoproteins and gets endocytosed by the peridermal cells from 10 minutes onwards. The time-lapse analysis along the z-axis confirmed that the tracer enters peridermal cells by apical endocytosis in the morphants (Figure S4).

To confirm that the material endocytosed from the apical domain is reaching the late endosomes or lysosomes, we followed the uptake of pHrodo Dextran. The fluorescence intensity of pHrodo Dextran increases in the acidic environment. After 6 hours of incubation, the tracer was found in the perinuclear compartments in the morphant peridermal cells confirming the acidic nature of these compartments whereas in wild type cells low signal for pHrodo Dextran was detected (Figure 3C,D). Such a low signal in wild type suggests that lysosomal degradation happens minimally in wild type peridermal cells possibly due to minimal routing of endosomes to this pathway. However, in the absence of *myoVb* function, a potential failure of recycling might cause the trapped apical endosomes to be targeted for degradation.

We further asked whether basolateral plasma membrane contributes to the formation of these late endosomal/lysosomal compartments. We used caveolin as a marker for endocytosis from the basolateral side. In the *myoVb* deficient peridermal cells, large aberrant caveolin labelled vesicles were observed tethered to the basolateral domain and in the cytoplasm at 27–30hpf (Figure 3E,F,H). In control embryos such vesicles were not observed. The Dextran uptake assay combined with caveolin staining revealed that Dextran is never localized to caveolin labelled vesicles (Figure S5A,B) further corroborating the finding that Dextran enters the peridermal cells from the apical side.

Beginning at 33hpf, we observed a third distinct phase of endocytosis in several peridermal cells selectively in *myoVb* morphants. It occurs just before the morphological cell rounding phenotype becomes apparent and is characterized by massive endocytic spurts. These spurts are marked with Caveolin and E-cadherin indicating their basolateral origin (Figure S5C–F). The time lapse microscopy analysis revealed that during these bursts the plasma membrane between two juxtaposed cells is endocytosed enabling the cells to change their neighbours, a process reminiscent of intercalation (Supplemental Movies 1, 2). The occurrence of these endocytic bursts decreases by 72hpf as the morphological phenotype subsides (Figure S5E–H).

To conclude, three distinct modes of endocytosis contribute to the formation of endosomes or lysosomes in *myoVb* deficient peridermal cells. In the first mode, the apical endosomes generated through fluid phase endocytosis are targeted to lysosomal degradation in the absence of *myoVb* function. The second mode comprises of caveolar endocytosis between 23–30hpf. The third mode, which begins after 33hpf, is characterized by endocytic spurts arising from the basolateral domain leading to cellular intercalation like events. Although fluid phase endocytosis was seen in wild type embryos, caveolar endocytosis and basolateral endocytosis was never observed in control siblings suggesting that these may be secondary consequences of alterations in cellular physiology under stress in the absence of *myoVb* function.

### Peridermal cells exhibit a reduced surface area in the absence of functional *gsp/myoVb*

**Figure 4:**
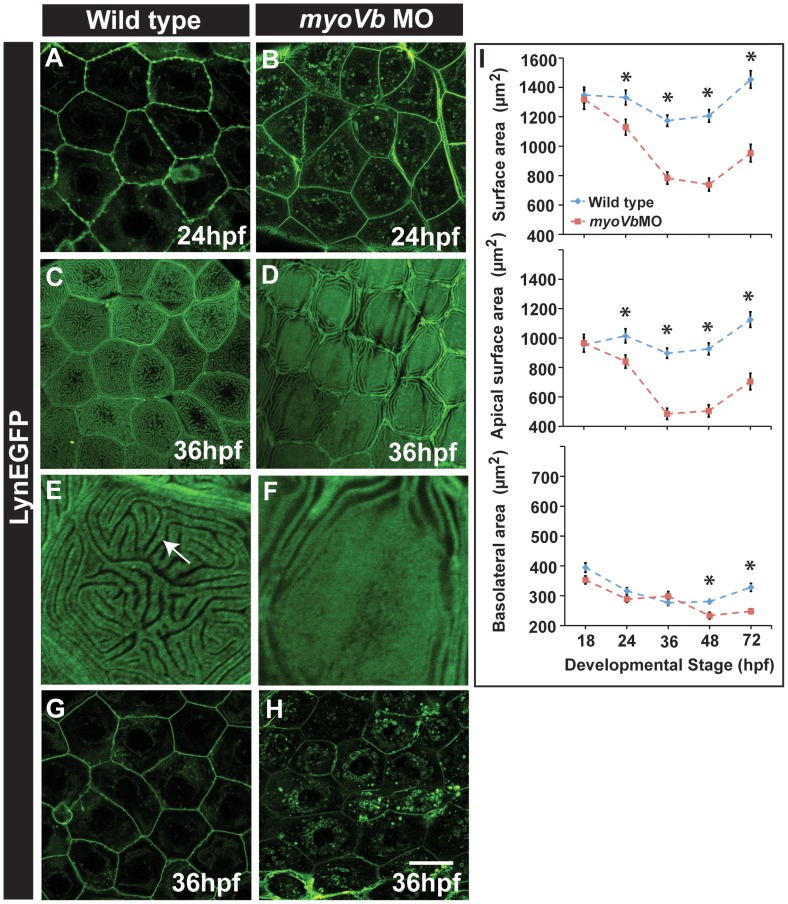
Effect of loss of *myosin Vb* function on membrane projections, cell size and cell surface area. LynEGFP staining in Tg(cldnB:lynEGFP) background revealed that membrane projections, which exist on basolateral side in wild type peridermal cells at 24hpf (A) are absent in *myoVb* morphants (B). Furthermore, apical microridges, present in wild type peridermal cells (C), are absent in *myosin Vb* morphants (D). E, F are digitally zoomed images of cells in ‘C’ and ‘D’, respectively. As compared to wild-type peridermal cells (G), morphant cells are smaller in size (H) at 36hpf. Quantification of cell surface area (I) reveals that total and apical surface area decreases substantially in morphants as compared to wild-type peridermal cells. The decrease in basolateral surface area is evident only after 48hpf. The error bars in ‘I’ represent standard errors of the mean whereas asterisks in ‘I’ indicate that the means are significantly different (student's t test p≤0.05). Arrow indicates an apical microridge in a peridermal cell. Scale bars correspond to 20 µ in A–D and G,H.

Our data indicate that in the absence of functional Myosin Vb, endosomes generated from apical and basolateral plasma membrane domains accumulate in the cytoplasm. We further investigated the effects of perturbed plasma membrane homeostasis on the epidermis. Endocytosis of the membrane components, followed by failure in recycling or membrane biogenesis, should lead to a decrease in the cell surface area. To accommodate the cytoplasmic volume, peridermal cells would initially unfold the membrane sequestered in the cellular projections and will eventually round up or bulge out leading to the *goosepimples* phenotype. We first analysed the membrane projections in the basolateral domain of the peridermal cells using cldnB:lynEGFP line. Fixed preparations at 24 hpf and in vivo live imaging between 18–26 hpf revealed numerous membrane ruffles in the basolateral domain in wild type embryos. In contrast, in the *myoVb* morphant larvae, membrane ruffles diminished over time and membranes appeared smooth and thin by 24 hpf with concomitant increase in the perinuclear vesicular pool (Figure 4A,B; Supplemental Movies 3, 4). In wild type periderm the micro-ridges showed random distribution all over the apical domain at 36hpf. In contrast, in the *gsp* mutants/*myoVb* morphants a large central region devoid of microridges was evident in the apical domain and ridges were more restricted to the peripheral region (Figure 4C–F).

Since there was a clear effect on apical micro-ridges and the membrane ruffles in *myoVb* deficient larvae, we further analysed the cell size and cell surface area. This was achieved by confocal imaging of E-cadherin- a basolateral marker- and lynEGFP stained peridermal cells followed by integration of the surface area of each con-focal section along the Z-axis. At 36hpf, the morphant peridermal cells exhibited smaller sizes as compared to the control embryos (Figure 4G,H). The systematic time course analysis revealed that the total surface area in *myoVb* morphants decreases by more than 30% between 18–48 hpf followed by a marginal increase between 48–72hpf (Figure 4I). The effect is more pronounced on the apical membrane domain as compared to the basolateral domain (Figure 4I).

Thus, the surface area of the peridermal cells shrinks in the absence of *myoVb* function presumably due to defects in plasma membrane homeostasis. The apical domain shrinks at a higher rate than the basolateral, suggesting that Myosin Vb has a preferential association with apical domain-directed traffic.

### Epidermal integrity is maintained by a dynamic equilibrium of cell number and cell size

**Figure 5:**
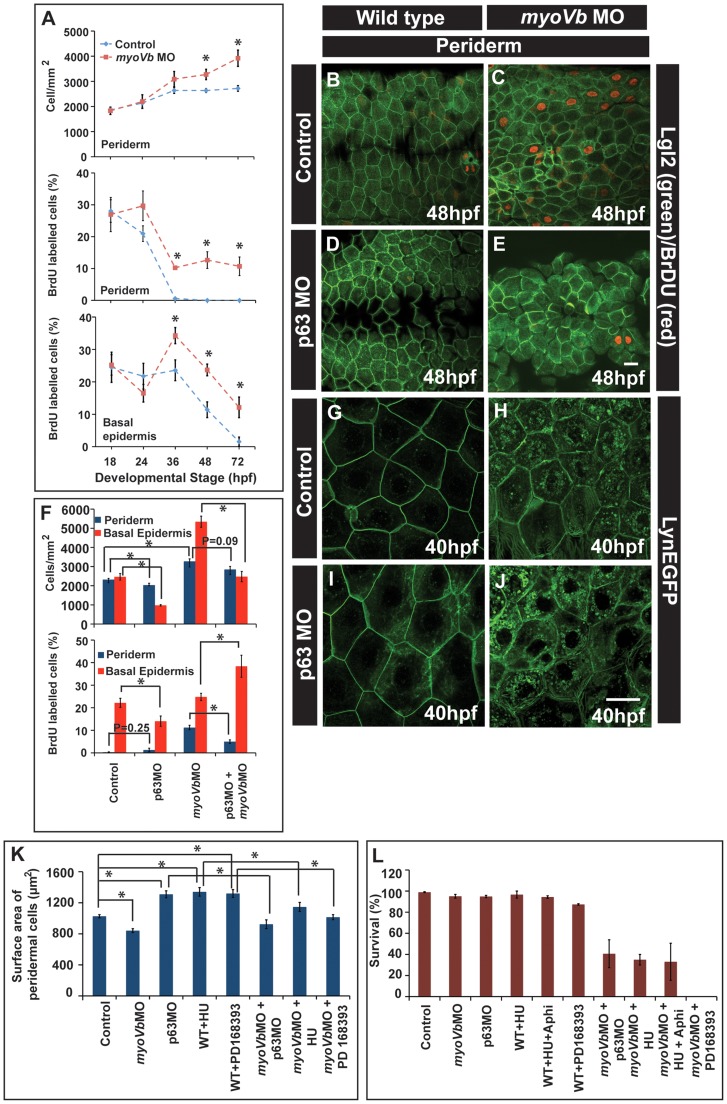
Two-way compensatory mechanism in zebrafish embryonic epidermis. Estimation of peridermal cell density and proliferation index in periderm and basal epidermis using BrdU labelling (A). Note the increase in number of peridermal cells per unit area, which is consistent with increase in the proliferation in the peridermal and basal epidermal cells beyond 24 and 36hpf, respectively. Lgl2 and BrdU labelling of the peridermal cells at 48hpf in wild type (B), *myoVb* morphant (C), *p63* morphant (D) and *p63*,*myoVb* double morphant (E). Quantification (F) of cell densities and proliferation indices by BrdU labelling in periderm and basal epidermis under genetic conditions represented in B-to-E. The lynEGFP staining revealed that as compared to wild type (G) the cells are smaller in *myoVb* (H) and larger in *p63* (I) morphants and comparable in *p63,myoVb* double morphants (J). Quantification of total surface area of a peridermal cell (K) at 40 hpf (for p63), 48 hpf (for PD 168393) and 50hpf (for HU) and percent survival (L) at 48hpf (for p63 and HU+Aphi), 58 hpf (for PD 168393), 74hpf (for HU) under various genetic conditions and treatments mentioned along the X-axis. Since control and *myoVb*MO conditions repeated in every treatment mentioned in (K), the data for these two was pooled to estimate the average. The square brackets indicate the comparison whereas asterisk indicates that the differences are statistically significant (students t test, p<0.05). The error bars represent the standard error of the mean. Scale bars in E and J correspond to 20 µ in B–E and G–J, respectively.

The surface area of the peridermal cells decreases in the absence of *myoVb* function. The peridermal cells appear smaller in the *gsp* mutant even at 7dpf (Figure S3G,H). Interestingly, the visible cell rounding phenotype of the *gsp* mutant/*myoVb* morphant phenotype subsides by 72hpf. Besides, there are no obvious breaches in the epidermal integrity. We therefore asked whether the decrease in cell size is compensated by the increase in cell number in *myoVb* morphant/mutant embryos and larvae to maintain epidermal integrity. Indeed, we observed that beyond 36hpf the number of peridermal cells per unit area is higher in the *myoVb* morphants as compared to wild type (Figure 5A). We further used a combination of BrdU incorporation and Lgl2 staining followed by confocal microscopy analysis to determine the proliferation status in *myoVb* morphants. BrdU analysis revealed more cell proliferation in both the periderm and basal epidermis of the morphants as compared to the wild type embryos and larvae (Figure 5A–C). In wild type embryos, proliferation in the periderm is minimal after 36hpf whereas in Myosin Vb deficient embryos and larvae the periderm retains a higher proliferative potential (Figure 5A). To rule out the possibility that cell death rather than reduced peridermal cell size is triggering the proliferation response, we performed TUNEL assay on the morphant embryos at 48hpf. Out of 8 larvae analysed by confocal microscopy, 5 did not show any apoptotic nuclei in the head periderm where the phenotype is the strongest (Figure S6A, B). The remaining 3 showed only 1–3 apoptotic nuclei suggesting that the cell death is minimal in the absence of Myosin Vb function (Figure S6C).

As the cell size decreases, the epidermis compensates by increasing the number of cells. However, it was not clear whether the converse is true. Furthermore, it was also not clear whether the increased epidermal proliferation in the *myoVb* morphants is indeed a compensatory response, essential for survival. To analyse this, we adopted three strategies: In the first, we knocked down the function of *delta p63*- a gene, which is essential for maintenance of the proliferative status in the epidermis, and in the second, we treated zebrafish embryos with inhibitors of cell proliferation- hydroxyurea and aphidicolin. As our third strategy, we inhibited EGF signalling in zebrafish larvae using a specific inhibitor PD168393 [62].

The number of cells in S-phase was decreased in *p63* morphants leading to reduced density of peridermal cells in wild type as well as Myosin Vb deficient embryos (Figure 5B–E, F). Similarly, phospho-histone H3 staining revealed that treatment with hydroxyurea resulted in decreased proliferation of basal as well as peridermal cells (Figure S7A–H,I). PD168393 treatment, starting at 18hpf, resulted in decrease in nuclear phosphorylated ERK (pERK) and concomitant reduction in the peridermal proliferation by 30hpf as assessed by BrdU incorporation (Figure S8A1–H). The effect of PD168393 treatment on proliferation was more profound in the periderm as compared to the basal epidermis (Figure S8M). The cell size analysis revealed that in *p63* morphants as well as in hydroxyurea and PD168393 treated embryos, cells were bigger and had significantly larger surface area as compared to control embryos (Figure 5G,I,K; Figure S7J,K,N,O; Figure S8I,J). The depletion of Myosin Vb in *p63* morphants or PD168393 or hydroxyurea treated embryos led to a decrease in cell sizes and surface area making them comparable to wild type peridermal cells (Figure 5G–J,K; Figure S7J–Q; Figure S8I–L).

At this stage, it is not clear how the loss of p63, which essentially acts in the basal epidermis, lowers peridermal cell number in the wild type and dampens the proliferation in the *myoVb* morphant periderm. One possible explanation is the involvement of a trophic factor- secreted by basal epidermal cells under the control of p63- that regulates peridermal proliferation. Intriguingly, p63 deficiency resulted in an increase in proliferation in the basal epidermis of the *myosin Vb* morphants (Figure 5F). The reason for this increase is not clear. However, this increased proliferation did not increase the cell density in the double morphants beyond wild-type level, reflecting its late onset.

There was a significant increase in lethality during 40–60 hpf in *myoVb* morphants injected with p63 morpholino or treated with hydroxyurea+aphidicolin or PD168393 (Figure 5L). In fact, none of the PD168393 treated morphants survived beyond 60hpf. The *myoVb* morphants treated with just hydroxyurea survived up to 72–75hpf and allowed us to investigate the effect of loss of cell proliferation on the peridermal cell morphology in *myoVb* morphants. The hydroxyurea treated *myoVb* morphants exhibited a rough epidermis phenotype along with misshaped peridermal cells at 72hpf in contrast to *myoVb* morphants alone, which recover by 72hpf (Figure S7N–U).

**Figure 6:**
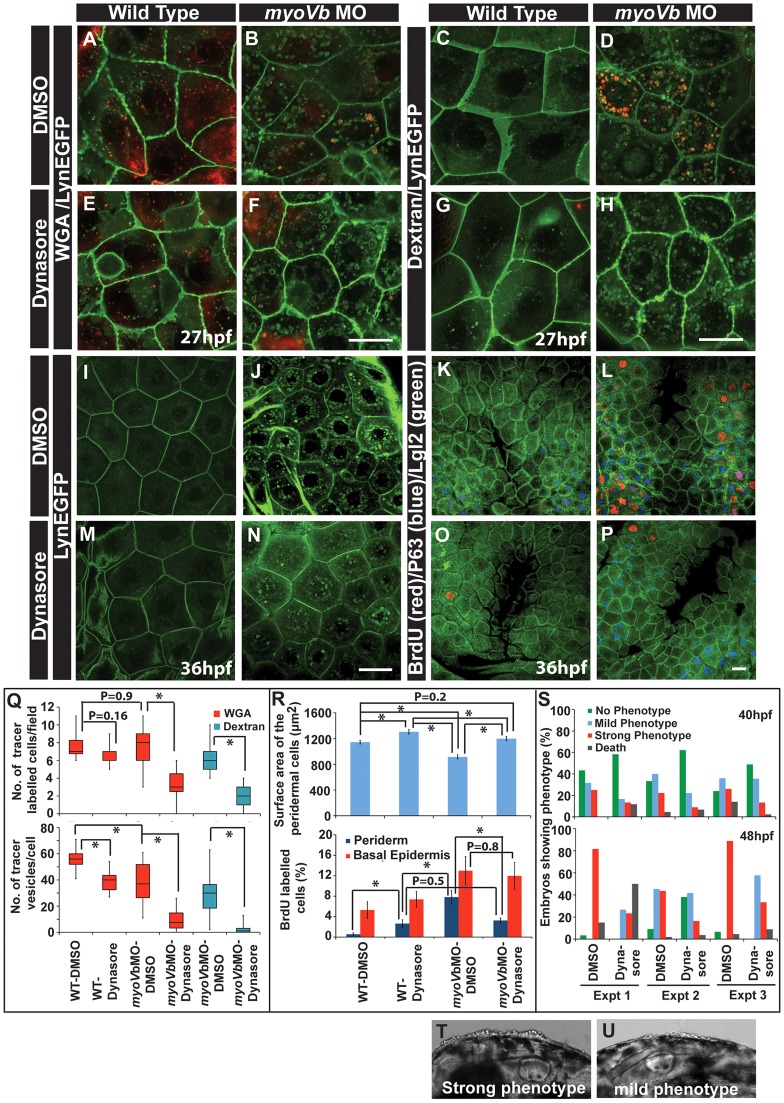
Reduction in endocytosis mitigates the cellular and morphological phenotype in Myosin Vb deficient embryos. Endocytosis of tracers WGA (A,B,E,F) and Dextran (C,D,G,H) in wild type (A,E, C,G) and *myoVb* morphant embryos (B,F,D,H) treated with DMSO (A,B,C,D) and dynasore (E,F,G,H) in the LynEGFP background. The dynasore treatment decreases endocytosis of both WGA and Dextran. Note that in wild type larvae there is hardly any dextran uptake indicating low rate of fluid phase endocytosis. As compared to wild type, WGA uptake appears less in the morphant. The reason being that the mucous layer is reduced in the mutants. The LynEGFP staining (I,J,M,N) in wild type (I,M) and *myosin Vb* morphants (J,N) treated with DMSO (I,J) and dynasore (M,N). Cell proliferation analysis using BrdU incorporation (K,L,O,P) in wild type (K,O) and *myoVb* morphant (L,P) treated with DMSO (K,L) and dynasore (O,P) in LynEGFP background. Note the decrease in proliferation in the morphant periderm upon dynasore treatment. Quantification of WGA and Dextran (Q) uptake by peridermal cells in different genetic backgrounds and under different treatments mentioned along the X axis. The first graph shows the number of cells per field showing uptake of the tracers. The second graph represents the number of vesicles per cell. For this analysis only the cells showing tracer uptake were considered. Quantification of area and cell proliferation for genetic backgrounds and various treatments shown along the X-axis (R). Quantification of embryos showing various degrees of phenotypic strengths (S) at 40 hpf (first graph) and 48 hpf (second graph). The morphant embryos were categorised in three phenotypic classes- strong, mild and no phenotype- based on the head periderm phenotype. The embryos classified as “no phenotypes” exhibited the decreased fin expansion and occasionally a few rounded up cells in the finfold. Bright field images showing strong (T) and mild (U) phenotypes. Scale bar in F, H corresponds to 10 µ in A,B,E,F and C,D,G,H, respectively whereas scale bars in N,P corresponds to 20 µ in I,J,M,N and K,L,O,P, respectively. Square brakets and associated aseterisks in Q and R represent the comparison by T-test and significant difference (P≤0.05), respectively.

To confirm that the cell size is linked with the proliferative status, we asked whether the peridermal cell proliferation reduces in *myoVb* morphants upon decrease in endocytosis. We reasoned if the endocytosis is inhibited, the cell surface area would be maintained in the *myoVb* morphants leading to mitigation of the proliferation phenotype. We used dynasore, a chemical inhibitor, to block the dynamin dependent endocytosis [63]. In the presence of dynasore there was a considerable decrease in endocytosis in the Myosin Vb deficient embryos as revealed by the uptake of two tracers, dextran and WGA, and by reduction in the number of Rab 5 labelled endosomes (Figure 6A–H, Q; Figure S9A–E). Although control *myoVb* morphants showed presence of dextran containing vesicles in the peridermal cells upon 4 hr incubation, there was no appreciable uptake of dextran by the wild type cells suggesting increased fluid phase endocytosis in the morphants (Figure 6C,D). The apparent decrease in WGA uptake by peridermal cells in the *myosin* morphants as compared to wild type embryos is presumably due to reduced binding of WGA to the apical domain (Figure S9F–H). This might be a consequence of decreased levels of surface mucous- to which WGA binds - in the Myosin Vb deficient embryos (Figure S3A, B). Since endocytosis was significantly reduced in the presence of dynasore, we asked whether there is any effect on the cell size and proliferation. Indeed, the area estimation revealed that as compared to untreated larvae, dynasore treated wild type and morphant embryos showed significant increase in the cell surface area (Figure 6I,J,M,N, R). Besides, there was a clear and specific decrease in cell proliferation in the periderm of the morphants but not in the basal epidermis confirming the link between peridermal cell size and proliferative status (Figure 6K,L,O,P,R). Interestingly, we also observed a visible effect on the strength of the phenotype upon dynasore treatment. While several of the untreated morphant embryos showed a stronger phenotype at 48 hpf (Figure 6T), dynasore treatment resulted in an increase in the number of embryos showing milder phenotypes (Figure 6U). A systematic quantification at two different time points, 40 and 48 hpf (N = 3), revealed a delayed onset as well as reduction in the strength of the phenotype in dynasore treated morphants (Figure 6S).

To conclude, our data suggest that the periderm maintains a dynamic balance between cell number and cell size. A decrease in cell size is balanced by an increase in cell number and vice versa. The *myoVb* function is essential to achieve an appropriate peridermal cell size in the absence of cell proliferation. Reduction in cell proliferation in the periderm of *myoVb* morphants leads to increased lethality presumably due to the compromised recovery of the tissue architecture. Furthermore, the mitigation of the morphological phenotype upon dynasore treatment suggests that the endocytosis is causally linked to the acquisition of cell rounding phenotype in Myosin Vb deficient embryos.

## Discussion

The maintenance of cell size in the epidermis and its role in tissue homeostasis has remained unclear so far. Here, we show that *gsp/myoVb* function is essential for the maintenance of peridermal cell size. By analysing *gsp/myoVb* mutant and morphant embryos, we have unravelled the importance of cell size in the maintenance of epidermal homeostasis during development.

The *myoVb* gene encodes an unconventional myosin motor, which is involved in cellular transport along the actin cables. It has been shown to interact with Rab8a, Rab10 and Rab11a and associate with the plasma membrane recycling systems [21], [22], [23], [24], [25]. In fact, its function has been shown to be essential for recycling of various receptors[26], [27], [29], [30], [31], for apical secretion [54] and for transport [32], [33], [34]. In the Drosophila genome, a single *myosin V* gene, *didum*, is shown to be essential for Rhodopsin transport in photoreceptor cells, for apical secretion in epithelial tubes and for the posterior localisation of *oskar* mRNA in the oocyte [38], [39], [40], [41]. Thus, although the functions of *didum* and *myoVb* have been well described in transport of various cargoes, the effect of loss of *myoVb* function on tissue architecture has not been investigated so far. Such an effect on the entire tissue could be due to changes in the physical parameters such as cell shape or size, as a consequence of perturbed endosomal transport, and may not be due to misrouting of a particular receptor or membrane component.

**Figure 7:**
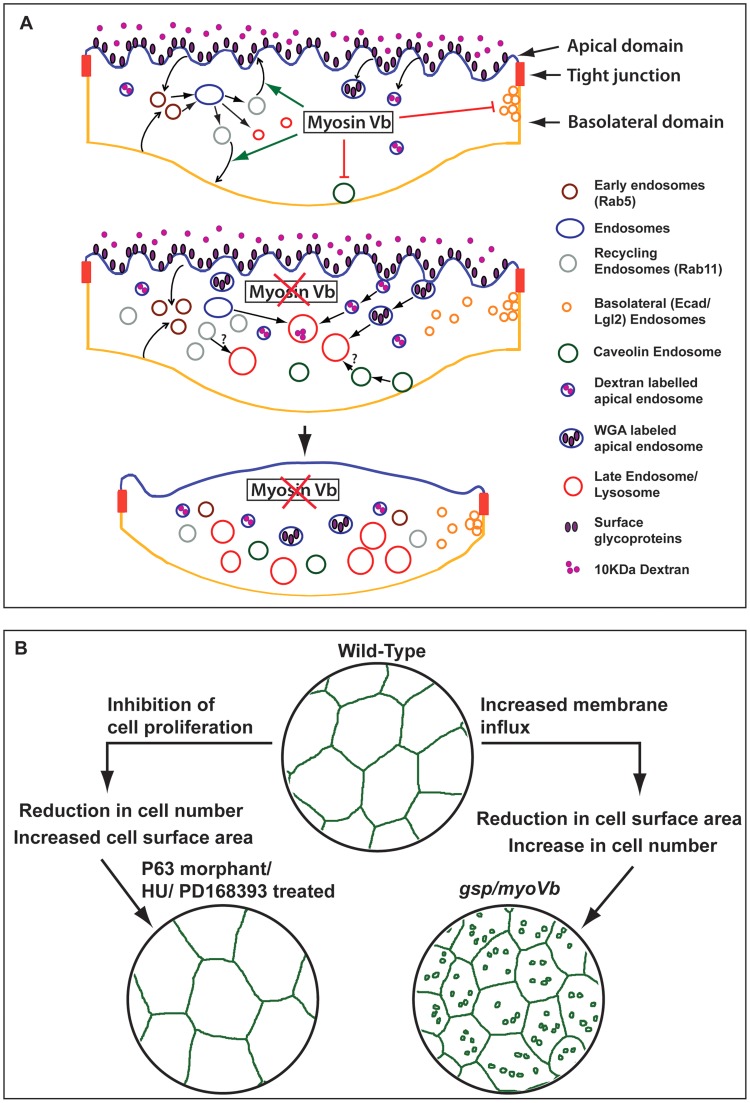
Schematic representation of *myosin Vb* function in the epidermis and its link with tissue homeostasis. In wild-type peridermal cells the endocytosis and recycling is balanced maintaining the plasma membrane homeostasis (A). In Myosin Vb deficient embryos, there is increased endocytosis and reduction in recycling leading to accumulation of endosomes, which are targeted for degradation in lysosomes (A). As a consequence the plasma membrane homeostasis is perturbed leading to shrinkage of cell surface area, which results in loss of membrane projections and in cell-bulging (A). Reduction in cell surface area in *gsp/myoVb* is compensated by increase in peridermal cell number. In contrast decrease in cell number results in bigger cells in the periderm (B). Epidermis maintains the dynamic balance between cell size and cell number.

Cells actively regulate their surface area, which directly depends upon the amount of plasma membrane [64]. In secretory cells, compensatory endocytosis is involved in retrieving the additional membrane added during exocytosis [65], [66]. Umbrella cells in urinary bladder undergo repeated cycles of expansion and shrinkage, which are regulated by concomitant Myosin Vb dependent exocytosis and dynamin dependent endocytosis, respectively [19], [35], [67]. We show that the *myoVb* function is essential for the maintenance of the cell surface area in zebrafish epidermis. Our data - based on Dextran, WGA uptake and labelling of various endosomal and lysosomal compartments - suggest that during embryogenesis wild-type peridermal cells exhibit significant endocytic and recycling activity with minimal lysosomal degradation. The total surface area per cell is maintained between 1200–1500 µm^2^ in the wild type periderm, suggesting that the retrograde (endocytic) membrane flux is balanced by the anterograde (recycling/biogenesis) flux (Figure 7A). In the *myoVb* deficient embryos the increase in Rab5, Rab11 and Rab7 labelled endosomes indicates enhanced endocytosis, possible reduction in recycling and accumulation of late endosomes. This increased retrograde flux in *myoVb* morphants is consistent with the role of Myosin Vb as an effector for Rab GTPases Rab8a, Rab10 and Rab11a, which are involved in membrane recycling or biogenesis pathways [23]. Intriguingly, there is also an increase in caveolin endocytosis and basolateral endocytosis; both may be secondary consequences of mechanical or physiological stress. The basolateral endocytic spurts are associated with cell rearrangements, which might help to counteract local stress arising due to cell shrinkage in *myoVb* morphants. Thus, in Myosin Vb deficient peridermal cells the retrograde membrane flux is much more than the anterograde flux. This results in imbalance of plasma membrane homeostasis, leading to shrinkage of cell surface area (Figure 7A). Consistently, the reduction in the retrograde flux by partial inhibition of the endocytosis results in restoration of the cell size in Myosin Vb deficient cells.

A recent report suggests that the size of neurons increase because of the failure of PTEN transport to the plasma membrane in the absence of both *myosin Va* and *myosin Vb* [37]. This is in contrast to our finding that peridermal cells shrink as a consequence of perturbed membrane homeostasis in the absence of Myosin Vb function. At present it is difficult to resolve this discrepancy but it is possible that the loss of PTEN from the plasma membrane overrides endocytosis, endosome accumulation and degradation in the neurons. This study does not report the effect of loss of Myosin V function on endosomal accumulation. It appears that the effect of the loss of motor function on cell size may be tissue and context- developing versus differentiated- dependent.

The *gsp/myoVb* mutant provides an ideal platform to study the effect of dysregulation of plasma membrane homeostasis on cell size, tissue architecture and development of the epidermis. In the *myoVb* morphants when the cell surface area decreases, the peridermal cells, which otherwise become post-mitotic, retain their division potential for a longer time. On the other hand, when cell proliferation is blocked, the cells increase their surface area to compensate for the lower number of cells in the tissue. Thus, in the epidermis cell number and cell size is regulated in such a way that a loss in one would be compensated by a gain in the other (Figure 7B). Such a two-way compensatory mechanism might be useful in maintaining epidermal architecture in the absence of either proliferation or cell expansion and under physical assaults, which lead to sudden loss of cells. Indeed, neither *gsp/myoVb* mutations nor inhibition of epidermal proliferation alone is lethal during 48–72hpf in zebrafish. However, inhibiting cell proliferation as well as *myoVb* activity leads to increased lethality presumably due to the effect on the epidermal architecture, which is evident in hydroxyurea treated *myoVb* morphants.

How does decrease in cell size trigger the proliferation? The decrease in cell surface area by increased inward membrane flux must generate an inward pull within each cell. Since peridermal cells are coupled to each other through cellular junctions, this inward pull will generate tension across the cell-cell contacts, which are mediated by adherens junctions. It has been shown that the increased mechanical stress leads to cadherin dependent sustained increase in cell proliferation [68]. Furthermore, pathways such as Hippo and Rho GTPase signalling may act as downstream effectors of mechanical stress to obtain increase in the proliferation [69], [70], [71]. It seems unlikely that *myoVb* deficiency directly activates proliferation independent of its effect on peridermal cell size, because the proliferative response is highly regulated and compensatory in nature and can be mitigated by dynasore treatment. One would expect a neoplastic response if a signalling pathway has gone awry in the absence of *myoVb* function. Indeed, it has been shown that activation of EGF signalling in *pen/lgl2* mutant leads to a cancer like phenotype in the zebrafish epidermis [62]. The link between the cell size and proliferation may not be specific to the *gsp/myoVb* mutant. We have identified at least two more genetic conditions, a zebrafish mutant called *romeharsha* and the previously published *clint1* loss of function scenario [72], which exhibit a vesicular accumulation phenotype similar to *gsp*. Both show reduced surface area and increased proliferation in the periderm (Phatak, et al., unpublished). We propose that plasma membrane homeostasis, in general, is linked with cell size regulation and cell proliferation in the periderm. The basal cell proliferation in *gsp/myoVb*, however, does not seem to be linked with peridermal cell size or proliferation as it does not show dampening upon dynasore treatment. The reason for this increased proliferation in the basal epidermis is currently unclear and needs to be investigated further.

The increase in cell surface area in the absence of proliferation requires Myosin Vb activity. This raises a possibility that the Myosin Vb function is also important for membrane biogenesis in the periderm. Myosin Vb has been shown to be involved in membrane biogenesis in the neurons by interacting with Rab10 [36]. Although we observed significant reduction in the Dextran or WGA labelled endosomes arising from the apical surface in the *MyoVb* morphants upon dynasore treatment, the lynEGFP labelled vesicular bodies still persisted in the cytoplasm. It is plausible that some of these are arising due to dynamin independent endocytosis while some are biogenic vesicles, which are not targeted to the plasma membrane in the absence of *myoVb* function.

In Myosin Vb deficient embryos, all the peridermal cells exhibit the accumulation of vesicles and reduction in the cell surface area but only a few, especially over the head, round up. There are at least four possible reasons why cells would round up in the absence of Myosin Vb function: a) This might be a result of more mechanical stress experienced by the peridermal cells over the head as the brain ventricles are expanding. b) The peridermal cell rounding is linked with the cell division [20]. It has been shown that mitotic cells round up during their progression from interphase to metaphase due to the reduction in their surface area as a consequence of clathrin mediated endocytosis. As the division proceeds, cells regain their shape by recycling of the accumulated membrane components [20]. In Myosin Vb deficient cells the lack of membrane recycling may leave dividing cells arrested in rounded up morphology. c) Members of the Myosin superfamily such as non-muscle Myosin II have been shown to be involved in force generation and cell shape maintenance [73]. Thus, Myosin Vb may have a structural role to play in maintenance of the cell shapes and hence in its absence cells assume rounded up morphology under mechanical stress. d) It is reasonable to argue that the cells that round up are physiologically different from the rest of the epidermal cells and exhibit more endocytosis. In such highly endocytic cells, the loss of *myoVb* function will have severe consequences leading to a faster decrease in cell surface area. Under such a scenario, peridermal cells would round up to accommodate the cytoplasmic volume. Indeed, peridermal cells in the dorsal head are derived from the dorsal EVL [74], which overlaps with non-involuting endocytic marginal cells having higher endocytic activity during gastrulation [75]. Although we cannot negate the possibility of Myosin Vb functioning in the shape maintenance, our finding that the reduction in endocytosis mitigates the cell rounding phenotype in myosin morphants favour the possibility that the extent of endocytosis is a major cause of cell rounding up phenotype. The link between rounding up and cell division can be easily ruled out because upon blockade of cell proliferation we still observe rounded up peridermal cells in the Myosin Vb deficient embryos. Whether the reason for rounding up of head peridermal cells is mechanical or physiological, the importance of *myoVb* function in head periderm is underscored by the fact that these cells exhibit elevated levels of transcription of *myoVb* at 2dpf as shown by in situ hybridisation.

Insights into the function of Myosin Vb in epithelial tissues at the organismal level have been gained from studies done on human individuals suffering from microvillus inclusion disease. This disease is caused by mutations in *myoVb* and is characterized by perpetual diarrhoea [52]. It has been shown that in these patients inclusion bodies consisting of microvillar components accumulate in the cytoplasm resulting in microvillar atrophy [76]. Our analyses show an effect on the organization of the apical microridges, actin based structures similar to microvilli, in peridermal cells. Although we did not observe inclusion bodies that contain either actin or Ezrin, we observed increased Dextran vesicles at the apical cortex in the uptake assays indicating increased apical membrane retrieval in Myosin Vb deficient peridermal cells. Our ongoing analysis of the *gsp/myoVb* mutant gut suggests that the gut epithelial cells do exhibit inclusion bodies. The mutant larvae do not survive beyond 12–14 dpf presumably due to the cumulative effect on epidermis and gut functioning.

To summarise, we have unravelled a hitherto unknown function of *myoVb* in the vertebrate epidermis. We have shown that Myosin Vb regulated plasma membrane homeostasis is essential for the maintenance of peridermal cell size. We have further been able to show that the epidermis has the ability to compensate for the loss of cell surface area by increasing its cell number and vice versa. We predict that such a mechanism would be useful under circumstances, such as large wounds, wherein there is a sudden decrease in the cell number. Our study warrants analysis of the barrier function of the epidermis in human patients suffering from the microvillus inclusion disease.

## Materials and Methods

### Ethics statement

For zebrafish maintenance and experimentation, the guidelines recommended by the Committee for the Purpose of Control and Supervision of Experiments on Animals (CPCSEA), Govt of India, were followed. Institute Animal Ethics Committee (IAEC) approved the animal care procedures and protocols used in this study vide the sanction TIFR/IAEC/2013-3.

### Fish strains

Three mutant alleles of *goosepimples*, *gsp^NS042^*, *gsp^NG061^* and *gsp^AT21^* were used for the experiments. Tg(cldnB:lynEGFP) line [55] was used for live imaging experiments to visualize plasma membrane. Tg (h2afx: EGFP-Rab5c)^mw5^, Tg (h2afx: EGFP-Rab11a)^mw6^ and Tg (h2afx: EGFP-Rab7)^mw7^ lines [57] were used to ascertain the nature of vesicles in the peridermal cells. Microinjections were done in *albino*, Tuebingen and pet-shop derived local strain. For in situ hybridization embryos from *albino* strain were used.

### Mapping and positional cloning

Mapping and positional cloning was done as described previously [77], [78]. For sequencing, cDNA was amplified using gene specific primers, which were designed using accession XM_690697.3 (replaced by NM_001161632), and using proofreading DNA polymerases (Fermentas, Roche). To detect the mutation in splice donor/acceptor site flanking exon 10, the region was amplified from genomic DNA (prepared for mapping) by PCR using high fidelity DNA polymerases (Takara, Roche) and directly sequenced. To check for a possible *myoVb* duplication, genomic DNA was isolated from mutants and sibling using 50 mM NaOH treatment at 95°C for 10 minutes followed by neutralization by 1 M Tris-HCl pH 8.0 [79]. To identify the mutations, sequence analysis was performed using either Lasergene software from DNASTAR or CLC main workbench. Conserved domains were identified using Simple Modular Architecture Research Tool (SMART; http://smart.embl-heidelberg.de).

### Morpholino injections

Antisense morpholino oligos against *myoVb* and p63 [16] were obtained from Gene Tools, LLC (Eugene, Oregon). Two separate morpholinos along with their respective 5 base mismatch controls were tested for *myoVb*: splice site directed- 5′GATCTTCTATTACTGACCGAGTTGA3′ and control 5′GATGTTGTATTACTCACCCACTTGA3′ (used at 200 µM); and ATG morpholino- 5′ ACTTTCCAATATCCACAGACGCACT3′ and control 5′ ACTATGCAAAATCCAGACACGCACT3′ (at 200 µM). All the morpholino studies were conducted using the splice-site morpholino. p63 morpholino 5′CCCTAGTTTTCTTCCTTTTATCCCC3′ was injected at 50 µM. All injections were done at 1–2 cell stage.

### Immunostaining, Dextran, WGA and Lysotracker staining, Dynasore treatment

Immunostainings were done as reported previously [80]. Embryos or larvae were fixed in 4% PFA in PBS and kept overnight at 4°C, followed by methanol up-gradation and storage at −20°C for staining with anti-GFP (Torrey Pines Biolabs; TP401), monoclonal anti-GFP (Genei, Bangalore), monoclonal anti-E-Cadherin (BD Transduction Labs; 610182), anti-Caveolin1 (BD Transduction Labs; 610059), anti-Lgl2 [80], rat monoclonal anti-BrdU (Acris Antibodies; SM1667P), anti-phospho histone 3 (Millipore, 06-570), monoclonal anti-p63 (Chemicon; MAB4135) antibodies. Larvae were fixed in Dent's fixative (80∶20, Methanol: DMSO) overnight at −20°C for staining with anti-ZO-1 antibody (ZYMED Labs, Invitrogen; 61-7300). For Rab5 staining, embryos were fixed in 4% PFA in PEMTT (0.1 M PIPES, 5 mM EGTA, 2 mM MgCl2 · 6H2O, 0.1% TritonX-100, 0.1% Tween 20, pH 6.8) [81] without methanol post-fixation followed by anti-GFP antibody (Torrey Pines Biolabs; TP401). The antibody dilutions used were as follows: rabbit anti-GFP (1∶200), mouse anti-GFP (1∶40), anti-E-Cadherin (1∶100), anti-Caveolin1 (1∶100), anti-Lgl2 (1∶400), anti-BrdU (1∶50), anti-phospho histone 3 (1∶400), anti-p63 (1∶100), anti-Ezrin (1∶250), anti-ZO-1 (1∶100), Alexa 488 conjugated secondary antibodies (1∶250), Cy3 and Cy5 conjugated secondary antibodies (1∶750).

For staining of live embryos and larvae, working dilutions of Lysotracker Red DND-99 (Invitrogen; L7528), 10 kDa Alexa 546-conjugated Dextran (Invitrogen; D-22911) and pHrodo Dextran (Invitrogen; P10361) were made in E3 medium without Methylene Blue. The larvae were incubated at 29°C in 5 µM Lysotracker for 3 hours or in 20 µg/ml pHrodo Dextran for 6 hours before washing in E3 and mounting in Methyl Cellulose for imaging. For 10 kDa Alexa 546-conjugated Dextran, the larvae were incubated in 10 mg/ml Dextran for 3 or 9 hours, washed and mounted for live imaging or fixed in PFA for imaging. For blockade of endocytosis, Alexa Fluor 594 conjugated Wheat Gram Agglutinin (WGA) or Alexa Fluor 546 conjugated 10 kDa Dextran (both from Invitrogen) were used as tracers in the presence of 25 µM dynasore hydrate (Sigma, D7693). The dynasore treatment started at 20hpf. The embryos were incubated at 24hpf for 1 h for WGA or at 21hpf for 4 h for Dextran in the presence of dynasore at 29°C, followed by wash and chase for 2 h in E3 buffer in the presence of dynasore. DMSO was used as a vehicle control at the concentration of 3.25 µM. Experiments were staggered in such a way that imaging could be done at 27hpf.

Electron microscopy analysis was done as described earlier [80].

### In situ hybridisation

The primers were designed for amplification of specific regions for *myoVaa* (NM_001080959.2; bp 2880–3795), *myosin Vb* (NM_001161632.1; bp 2967–3866), *myoVc* (XM_686051.3; bp 2920–3720) using Primer3 (version 0.4.0) and cloned into pCR TOPO 2.1 or pCR TOPOII (Invitrogen). For RNA probe synthesis, templates were linearised and probes were synthesized using either T7 or SP6 RNA polymerase (Roche DIG RNA Labelling Kit). In situ hybridisations were performed as described [82] with a few modifications. For sectioning, embryos were post-fixed in 4% PFA after the completion of in situ hybridisation protocol and embedded in Epon. Sections were cut at 3 µ thickness using glass knives on Leica microtome, placed on a glass slide coated with 1% gelatin, counterstained with 4% eosin made in 90% ethanol and mounted in DPX. Sections were imaged on Zeiss ApoTome using Axiocam.

### Image acquisition and processing

Imaging of immunostainings was done mostly over the head (dorsal head periderm) using either the Zeiss LSM 510 Meta with Plan-apochromat 63×/1.40 oil or EC Plan-Neofluar 40×/1.30 oil objective or on Zeiss LSM 710 with Plan-apochromat 63×/1.40. An optical zoom of 1.5× zoom was used for most of the images. 1024 by 1024 image dimensions were used, with an averaging of 4. In situ hybridization stainings were imaged either on Zeiss SteREO Discovery using AxioCam or on OLYMPUS SZX12 with OLYMPUS Camedia c-5050 Zoom camera. Imaging of peridermal cells of dorsal head region at 48hpf was done on Zeiss Axioscope 2.0 with 20×/0.75 objective.

For time lapse live imaging, larvae were dechorionated, treated with MESAB (Sigma; A5040) and embedded in 0.2% Agarose in plastic dishes with cover slip bottoms. Imaging was done on Zeiss LSM 5Live with Achroplan IR 63×/0.95 W objective, installed with a CO2 and temperature controlled chamber. The capture rate was one frame per 3 minutes for Supplementary Movie 1, 2 and one frame per 6 minutes for Supplementary Movie 3,4. Live imaging of WGA uptake from the apical surface was done on Zeiss LSM 510 using 40×/1.30 oil objective with 2× digital zoom on embryos immersed in WGA followed by immediate mounting in 0.2% agarose without washes. The scanning involved 15 optical sections in Z-direction and total 13 scan cycles, each of the duration of 238 seconds.

For imaging phenotypes and for Dextran, WGA, Rab5 live imaging as well as Lysotracker experiments, larvae were embedded in 3% Methyl Cellulose gel. DIC imaging was done on Zeiss SteREO Discovery with AxioCam. Zeiss LSM 5Live was used for Lysotracker imaging. Zeiss LSM 710 with 63× Plan-apochromat objective/1.4NA with 2× Zoom was used for Dextran and WGA assays as well as for live imaging of Rab5 vesicles associated with dynasore treatment.

Either ImageJ or ZEN Light Edition 2009 was used for image processing and analysis.

### BrdU incorporation assay, inhibitor treatments, TUNEL staining

30–40 larvae were dechorionated and incubated in 6-well plates at 29°C in either 10 µM solution of BrdU in 2% DMSO in E3 or only 2% DMSO in E3 for 2 hours. They were given three 5 minutes washes with E3, followed by PFA fixation and methanol up-gradation. After downgrading to PBS and prior to processing for immunostaining, the fixed larvae were treated with 4N HCl for 20 minutes at room temperature and processed for immunostainings.

For Hydroxyurea treatment, working concentration of 50 mM was prepared using E3 without methylene blue. In case of combined treatment, 30 mM hydroxyurea and 150 uM aphidicolin was used. Final concentration of DMSO was adjusted to −0.05% v/v to increase the permeability of Aphidicolin. Both Hydroxyurea and combined treatment of Hydroxyurea+Aphidicolin were done at 24hpf on dechorionated embryos. The 1 mM stock of PD168393 (Merck Millipore 513033) was prepared in DMSO and used at 10 µM final concentration in 1% DMSO in E3 starting from 18hpf.

The TUNEL staining was performed using Promega Kit as per the manufacturer's instructions. Briefly, the embryos at 48hpf were fixed in 4%PFA, washed in PBT (0.1 m phosphate buffer and 0.8% triton X-100), incubated with TUNEL reaction mixture at 37°C followed by washes, DAPI staining and mounted in Glycerol for imaging.

### Cell surface area quantification

For measuring surface area of plasma membrane, morpholino injections were done in Tg(cldnB:lynEGFP) background and the larvae were fixed at different stages and stained with anti-GFP and anti-E-Cadherin antibodies to obtain total surface area and basolateral area, respectively. Confocal stacks were captured at a slice interval of 0.373 µM. To quantify surface area, cell outlines were traced in each slice by monitoring the Measure Stack plug-in of ImageJ. The perimeter in each slice was multiplied by the slice thickness and added along with the area of the first and the last slice to obtain an estimate of total surface area. To quantify the area of the micro-ridges, images were smoothened and thresholded such that edges of the ridges were neatly defined. This was followed by the Analyze Particle command to detect and measure edge lengths. Detection was monitored and aided by manual detection using Wand (tracing) tool wherever required. The perimeter of all the edges was summed up and multiplied with slice thickness and number of slices the ridges extend to in order to get an estimate of the area sequestered in the membrane folds. Five cells from five larvae were analyzed for each population. The apical area was determined by subtracting basolateral surface area from the total area.

### Cell, nuclei, vesicle counting and statistical analysis

Quantification of BrdU and phospho-histone labelled nuclei as well as total cells was done using Cell Counter plug-in of ImageJ. In case of BrdU labelling, five larvae were analyzed for each population for various developmental stages and eight larvae each were analyzed for *myoVb* and *p63* interaction analysis. For phospho-histone, 7–10 larvae were analysed for each treatment.

For counting of Rab11 vesicles, live imaging of 3 control and 3 morphant embryos was done at 30 hpf for 10 minutes. For each embryo vesicles were counted at 3 different time frames by making a maximum intensity projection of 4 z stacks surrounding the nuclear region. Each embryo thus yielded three data points obtained from 4–7 cells (based on nuclear count). The value for each cell was derived by dividing the total number of vesicles by the total number of cells. The average number of vesicles was estimated from 9 data points each for control and morphant embryos. For Rab 5 vesicles, fixed preparations of 6 embryos were used with E-cadherin as a counter-stain allowing estimation of vesicle number/cell for 5 cells per embryos.

For estimation of Dextran and WGA vesicles, live imaging of 12 control and 12 morphant (N = 3) embryos was done at 27–28 hpf. First level analysis involved counting cells showing uptake of tracer within the field. At the second level, from the cells showing the uptake, vesicles were counted along X-Z direction using tracer tool in Metamorph image analysis software. ImageJ was used for the quantification of WGA fluorescence at the apical surface. For analysis of Rab5 vesicles, after live imaging vesicles were counted from 13–15 cells belonging to 4–5 embryos at 28hpf using the Metamorph tracer tool as described above. The data was presented using Box and Whisker plot.

F-tests for equality of variance followed by pair-wise comparison of means by two-tailed Student's t-tests were conducted for most of the analyses and a significance level of 0.05 was used as cut-off. Microsoft EXCEL was used for statistical analysis and plotting graphs.

## Supporting Information

Figure S1 Bright field images of wild type (A) and *gsp* mutant (B) larvae at 78hpf. E-cadherin staining in wild type (C) and *gsp* mutant (D) at 48hpf reveals no apparent effect on shapes of basal epidermal cells. Myosin Vb cDNA sequencing from *gsp^NG061^* allele reveals that an exon is absent in the mutant transcript (E). Sequence comparison (F) indicates that conserved Rab11 binding sites (boxed amino acids) are present in zebrafish Myosin Vb. Representative images of 5 base mismatch ATG control morpholino (G), *myoVb* start-site/ATG morpholino (H), 5 base mismatch splice-site control (I) and splice site morpholino (J) injected larvae at 48hpf. Arrows in H and J point to the rounded up peridermal cells over larval head, which is a classic feature of *myosin Vb* loss of function phenotype.
(JPG)

Figure S2 PCR amplification of intron 10–11 and intron 17–18 from genomic DNA isolated from *gsp* mutant larvae (L1) shows bands of 2.4 kb and 288 bp sizes, respectively. Additional bands of 1.7 kb (for intron 10–11) and 371 bp (for intron 17–18) sizes are expected if two copies of *myosin Vb* are present at the genomic interval (A). Genomic sequencing of PCR product of 361 bp around the mutation in *gsp^NS042^* allele from wild type (+/+), heterozygous (+/−) and three mutant (−/−) embryos reveals that mutants do not show presence of “T” along with “A” which is indicative of the presence of second copy of *myosin Vb* gene (B). Expression analysis of *myosin Vb* (C), *myosin Va* (D) and *myosin Vc* (E) by in situ hybridisation (ISH) at 48hpf. Section of 48 h old larva stained for *myosin Vb* expression by ISH and counterstained by eosin (F). RT-PCR analysis (G) reveals that *myosin Va* and *Vc* transcripts (arrows) are maternally contributed whereas *myosin Vb* transcripts are not present at 2–4 cells stage. Please note that cDNA preparation from 48hpf old larvae were used as a positive control and actin primers are used to check the quality of cDNA prepared from different stages. Lane C in (A) shows size-control PCR product of 361 bp and marks the approximate position for the 371 bp band if the second copy would be present. Arrowheads in (F) indicate the ISH signal in the outermost peridermal layer of the epidermis whereas arrows indicate the staining at interphase between the epidermis and the brain. Asterisks in E indicate 1 kb marker band.
(JPG)

Figure S3 Transmission electron micrograph of the wild type (A, C) and *gsp* mutant (B, D) peridermal cells. At lower magnification wild type cells (A) exhibit the mucous layer (arrows in A) on the apical side, which is absent in the mutant cells (B). Besides, mutant cells exhibit large transparent vesicular bodies marked with asterisks (B). At higher magnification a few vesicles (arrows in C) and microridges (arrowheads in C) are seen in the wild-type peridermal cells. The mutant cells show accumulation of several smaller vesicles (arrows in D) but loss of microridges. LynEGFP staining in *gsp/myoVb* mutant (E) and *myoVb* morphant (F) show similar cellular phenotypes at 48 hpf. At 7 dpf, size of peridermal cells in the *gsp* mutant (H) is smaller than in wild type (G). Lysotracker staining in LynEGFP line in wild-type (I) and *myoVb* morphants (J) at 48hpf and overlay for the lysotracker and LynEGFP in WT (K) and *gsp* mutant (L) at 8 dpf. These stainings reveal accumulation of lysosomes in the morphant and mutant peridermal cells. Abbreviations- PE-Nu: peridermal cell nucleus; BE-Nu: Basal epidermal cell nucleus. Scale bar in B and D are equivalent to 1 µ whereas those in F, H and J are equivalent to 20 µ.
(JPG)

Figure S4 Live time-lapse analysis of WGA uptake in *myoVb* morphants in lynEGFP background across the indicated scan-cycles and z-sections. The arrowheads indicate formation and endocytosis of an apical vesicle. Note the disappearance of the vesicle in Z section −2 between 18.3 and 732.3 seconds. In section-5, this vesicle emerges as time progresses from 73.2 to 787.2 seconds. t = relative frame acquisition time.
(JPG)

Figure S5 Simultaneous labelling using LynEGFP, Alexa 546 conjugated Dextran and anti caveolin antibody in control (A) and *myoVb* (B) morpholino injected embryos. The caveolin and Dextran label do not co-localise suggesting that Dextran enters in the cytoplasm only from the apical side of the peridermal cells. Immuno-localisation of Caveolin and LynEGFP in wild-type (C) and *myoVb* morphant (D) embryos at 40hpf. Arrows indicate spurts of endocytosis in the morphants whereas arrowheads indicate internalised caveolin vesicles. Overlay for E-cadherin (red) and LynEGFP (E–H) reveals that the endocytic spurts in the morphants (arrows in F) are labelled for E-cadherin at 48hpf and the frequency of the spurts decreases by 72hpf (H). Scale bars in B and D are equivalent to 10 µ whereas in H is equal to 20 µ.
(JPG)

Figure S6 TUNEL (green, nuclear), LynEGFP (green, membrane label) and DAPI staining (blue) of wild type (A) and morphant embryos (B, C) at 36hpf. The arrow (C) indicates nuclear TUNEL staining in the *myoVb* morphant periderm. Scale bar corresponds to 20 µ.
(JPG)

Figure S7 LynEGFP (green), phosphohistone-3 (red) and DAPI (blue) staining in wild type peridermal cell (A) and basal epidermal cells (B); HU treated periderm (C) and basal epidermis (D); *myoVb* morphant periderm (E) and basal epidermis (F); HU treated morphant periderm (G) and basal epidermis (H). Quantification of mitotic indices (I) under genetic condition and treatments mentioned along the X-axis. In myosin morphants peridermal cells exhibit increased mitosis. HU treatment inhibits proliferation in the basal epidermis of wild type and in the basal epidermis as well as periderm of *myoVb* morphants. LynEGFP staining in periderm of wild type control at 52 and 72 hpf (J, N), HU treated wild type (K,O), *myoVb* morphant (L,P) and HU treated *myoVb* morphant (M,Q). Note increased cell size upon HU treatment in wild type larvae (K,O) and irregular peridermal cell shapes in the HU treated *myoVb* morphants (Q). DIC images of wild type control (R), HU treated wild type (S), *myoVb* morphant (T) and HU treated *myoVb* morphant larvae (U) at 72hpf. While the peridermal cell rounding phenotype over the head recovers by 72hpf in *myoVb* morphants, it persists in HU treated morphants (arrow in U). Scale bars in H and Q correspond to 20 µ in A–H and J–Q, respectively.
(JPG)

Figure S8 pERK (A1,B1,C1,D1) and pERK/LynEGFP overlays (A1,B2,C2,D2) in wild type (A1,A2,B1,B2) and *myoVb* morphants (C1,C2,D1,D2) treated with DMSO (A1,A2,C1,C2) and PD168393 (B1,B2,D1,D2) at 30hpf. Note the clear decrease in pERK in morphants treated with PD168393. Analysis of cell proliferation using BrdU (E,F,G,H) at 30hpf and cell size using lynEGFP line at 48hpf (I,J,K,L) in wild type (E,F,I,J) and *myosin Vb* morphant (G,H,K,L) treated with either DMSO (E,I,G,K) or PD168393 (F,J,H,L). In ‘H’ the few BrdU labelled nuclei of the basal epidermis are in focus at the periphery of the field. Quantification of cell proliferation in periderm and basal epidermis (M) in various genetic conditions and treatments shown across the X-axis. Scale bars in D2, L and H corresponds to 20 µ in A1–D2,I–L and in E–H, respectively. The square brackets and asterisk in (M) represent the comparisons by T-test and significant difference at (p≤0.05).
(JPG)

Figure S9 Live imaging of EGFP-Rab5 endosomes in wild type (A,C) and *myoVb* morphant embryos (B,D) treated with DMSO (A,B) and Dynasore (C,D) at 28hpf and their quantification (E). WGA labelling in wild type (F) and *myosin Vb* morphant embryos (G) at 28hpf. Quantification of the labelling intensities in wild type and morphant peridermal cells (H). Scale bars in D and G equals to 10 µ. The square brackets (in E, H) represent the comparisons and the asterisks indicate the significant difference (t test; P<0.01).
(JPG)

Movie S1 Time-lapse movie analysis of wild type periderm labelled with lynEGFP from 43 to 48 hpf.
(AVI)

Movie S2 Time-lapse analysis of *myosin Vb* morphant periderm labelled with lynEGFP from 36 to 41hpf.
(AVI)

Movie S3 Time-lapse movie of the lynEGFP labelled periderm in wild type starting from 18hpf for 7 hours.
(AVI)

Movie S4 Time-lapse movie of lynEGFP labelled *myosin Vb* morphant periderm from 18hpf for 7 hours.
(AVI)
