# Supplementary figures and images for: Myosin Vb Mediated Plasma Membrane Homeostasis Regulates Peridermal Cell Size and Maintains Tissue Homeostasis in the Zebrafish Epidermis
Source: PLoS Genet. 2014 Sep 18;10(9):e1004614. doi: 10.1371/journal.pgen.1004614 (PMC4169241; doi:10.1371/journal.pgen.1004614)

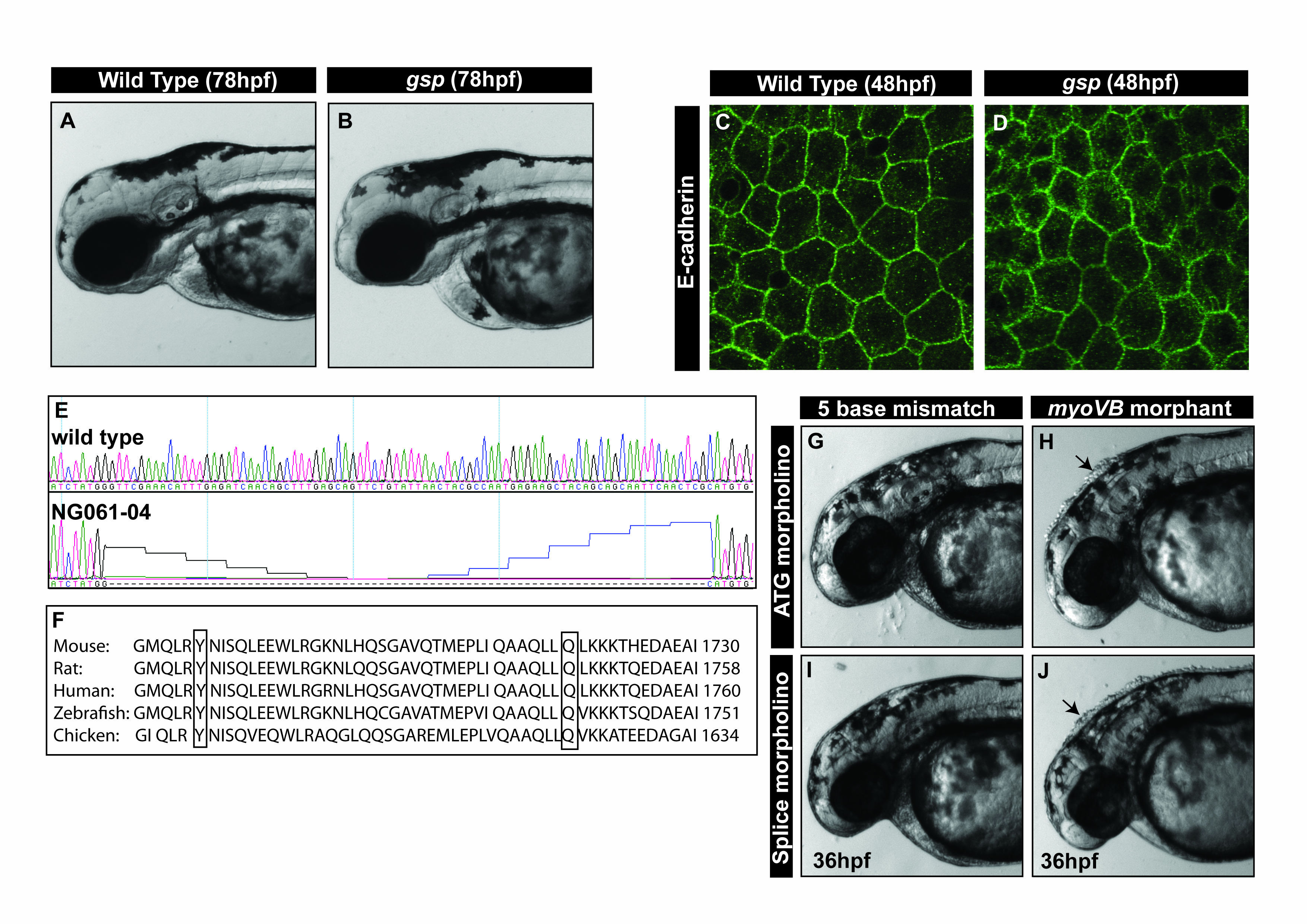

Supplement: Figure S1 — Bright field images of wild type (A) and gsp mutant (B) larvae at 78hpf. E-cadherin staining in wild type (C) and gsp mutant (D) at 48hpf reveals no apparent effect on shapes of basal epidermal cells. Myosin Vb cDNA sequencing from gspNG061 allele reveals that an exon is absent in the mutant transcript (E). Sequence comparison (F) indicates that conserved Rab11 binding sites (boxed amino acids) are present in zebrafish Myosin Vb. Representative images of 5 base mismatch ATG control morpholino (G), myoVb start-site/ATG morpholino (H), 5 base mismatch splice-site control (I) and splice site morpholino (J) injected larvae at 48hpf. Arrows in H and J point to the rounded up peridermal cells over larval head, which is a classic feature of myosin Vb loss of function phenotype. (JPG) [file pgen.1004614.s001.jpg]

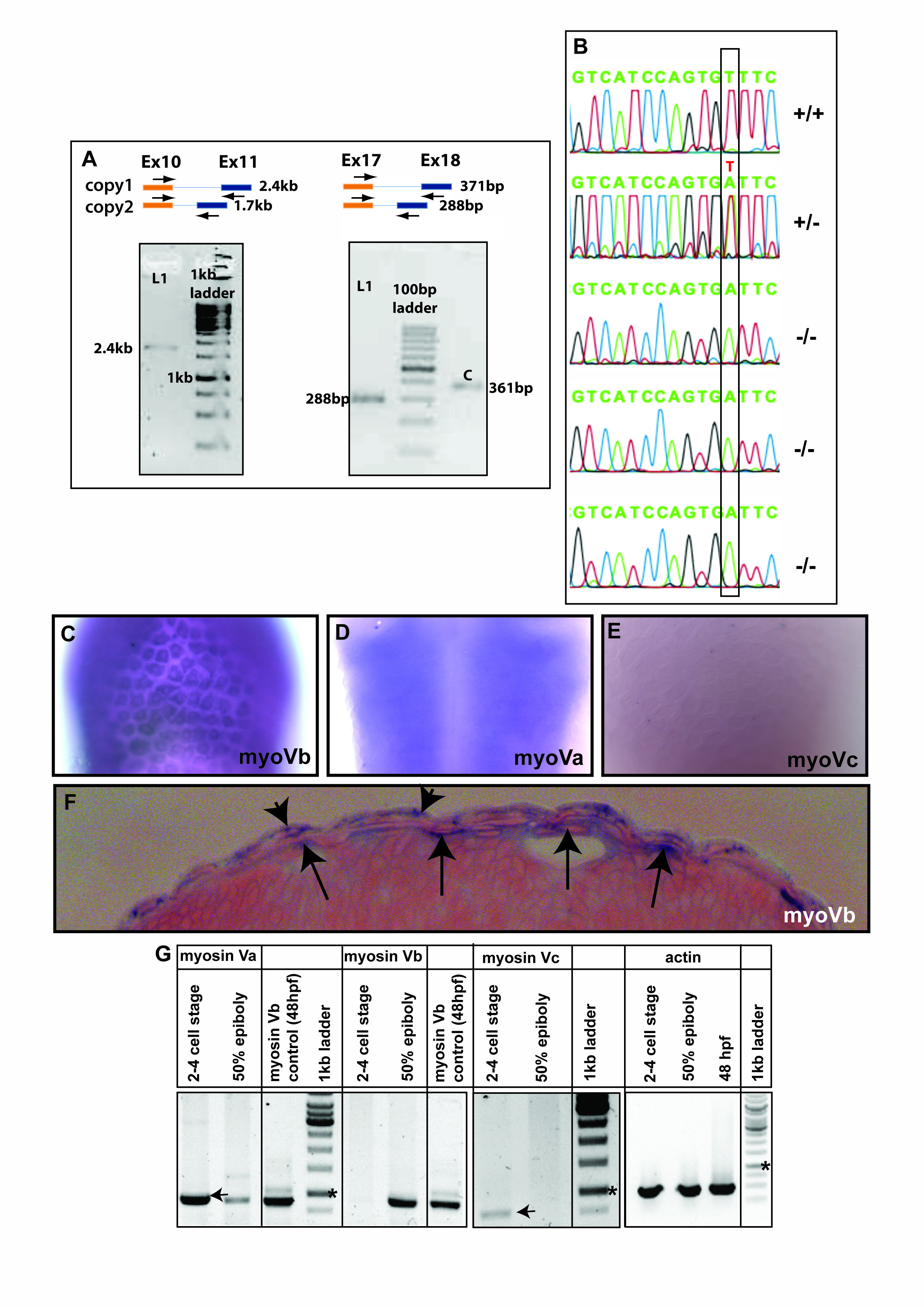

Supplement: Figure S2 — PCR amplification of intron 10–11 and intron 17–18 from genomic DNA isolated from gsp mutant larvae (L1) shows bands of 2.4 kb and 288 bp sizes, respectively. Additional bands of 1.7 kb (for intron 10–11) and 371 bp (for intron 17–18) sizes are expected if two copies of myosin Vb are present at the genomic interval (A). Genomic sequencing of PCR product of 361 bp around the mutation in gspNS042 allele from wild type (+/+), heterozygous (+/−) and three mutant (−/−) embryos reveals that mutants do not show presence of “T” along with “A” which is indicative of the presence of second copy of myosin Vb gene (B). Expression analysis of myosin Vb (C), myosin Va (D) and myosin Vc (E) by in situ hybridisation (ISH) at 48hpf. Section of 48 h old larva stained for myosin Vb expression by ISH and counterstained by eosin (F). RT-PCR analysis (G) reveals that myosin Va and Vc transcripts (arrows) are maternally contributed whereas myosin Vb transcripts are not present at 2–4 cells stage. Please note that cDNA preparation from 48hpf old larvae were used as a positive control and actin primers are used to check the quality of cDNA prepared from different stages. Lane C in (A) shows size-control PCR product of 361 bp and marks the approximate position for the 371 bp band if the second copy would be present. Arrowheads in (F) indicate the ISH signal in the outermost peridermal layer of the epidermis whereas arrows indicate the staining at interphase between the epidermis and the brain. Asterisks in E indicate 1 kb marker band. (JPG) [file pgen.1004614.s002.jpg]

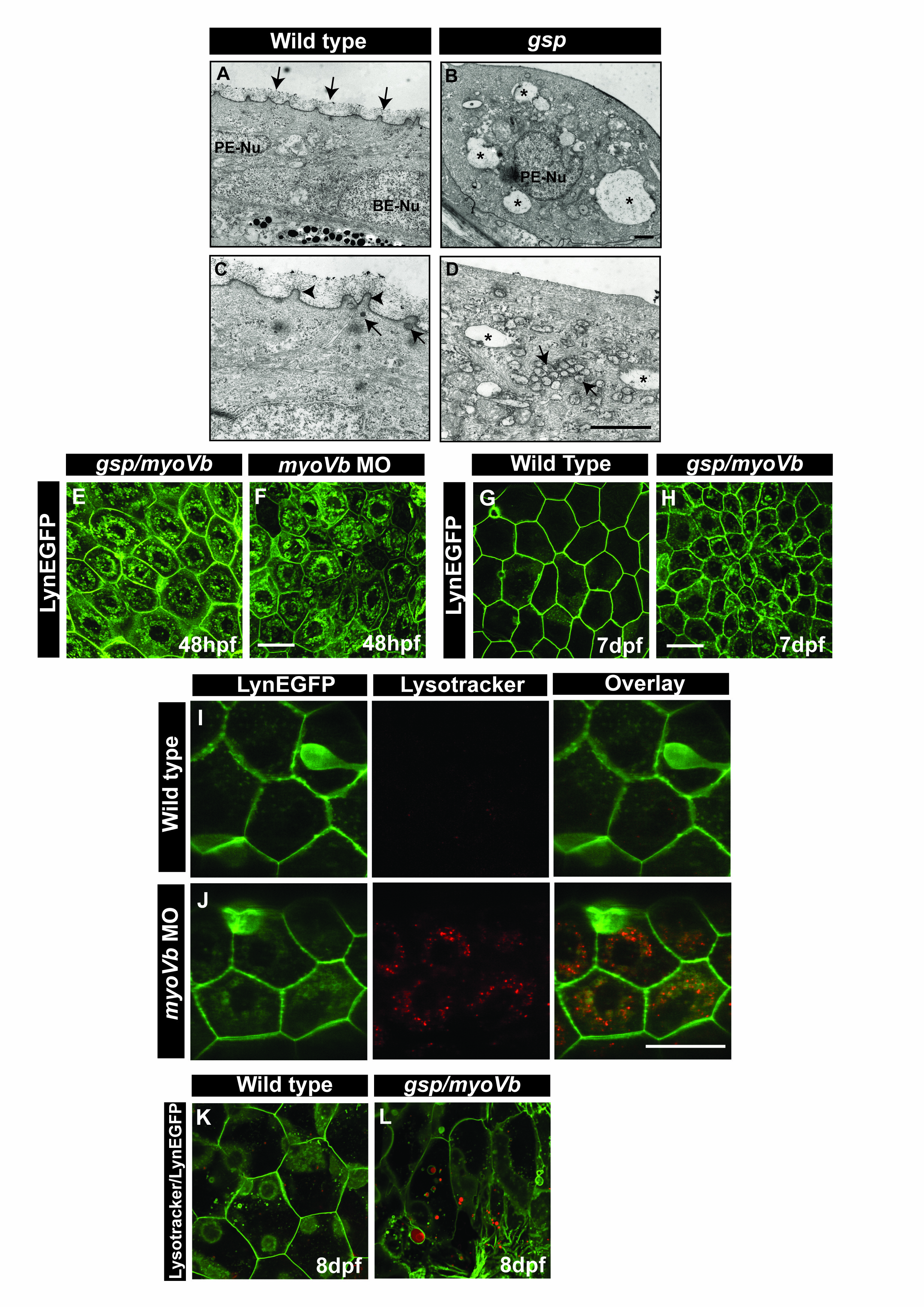

Supplement: Figure S3 — Transmission electron micrograph of the wild type (A, C) and gsp mutant (B, D) peridermal cells. At lower magnification wild type cells (A) exhibit the mucous layer (arrows in A) on the apical side, which is absent in the mutant cells (B). Besides, mutant cells exhibit large transparent vesicular bodies marked with asterisks (B). At higher magnification a few vesicles (arrows in C) and microridges (arrowheads in C) are seen in the wild-type peridermal cells. The mutant cells show accumulation of several smaller vesicles (arrows in D) but loss of microridges. LynEGFP staining in gsp/myoVb mutant (E) and myoVb morphant (F) show similar cellular phenotypes at 48 hpf. At 7 dpf, size of peridermal cells in the gsp mutant (H) is smaller than in wild type (G). Lysotracker staining in LynEGFP line in wild-type (I) and myoVb morphants (J) at 48hpf and overlay for the lysotracker and LynEGFP in WT (K) and gsp mutant (L) at 8 dpf. These stainings reveal accumulation of lysosomes in the morphant and mutant peridermal cells. Abbreviations- PE-Nu: peridermal cell nucleus; BE-Nu: Basal epidermal cell nucleus. Scale bar in B and D are equivalent to 1 µ whereas those in F, H and J are equivalent to 20 µ. (JPG) [file pgen.1004614.s003.jpg]

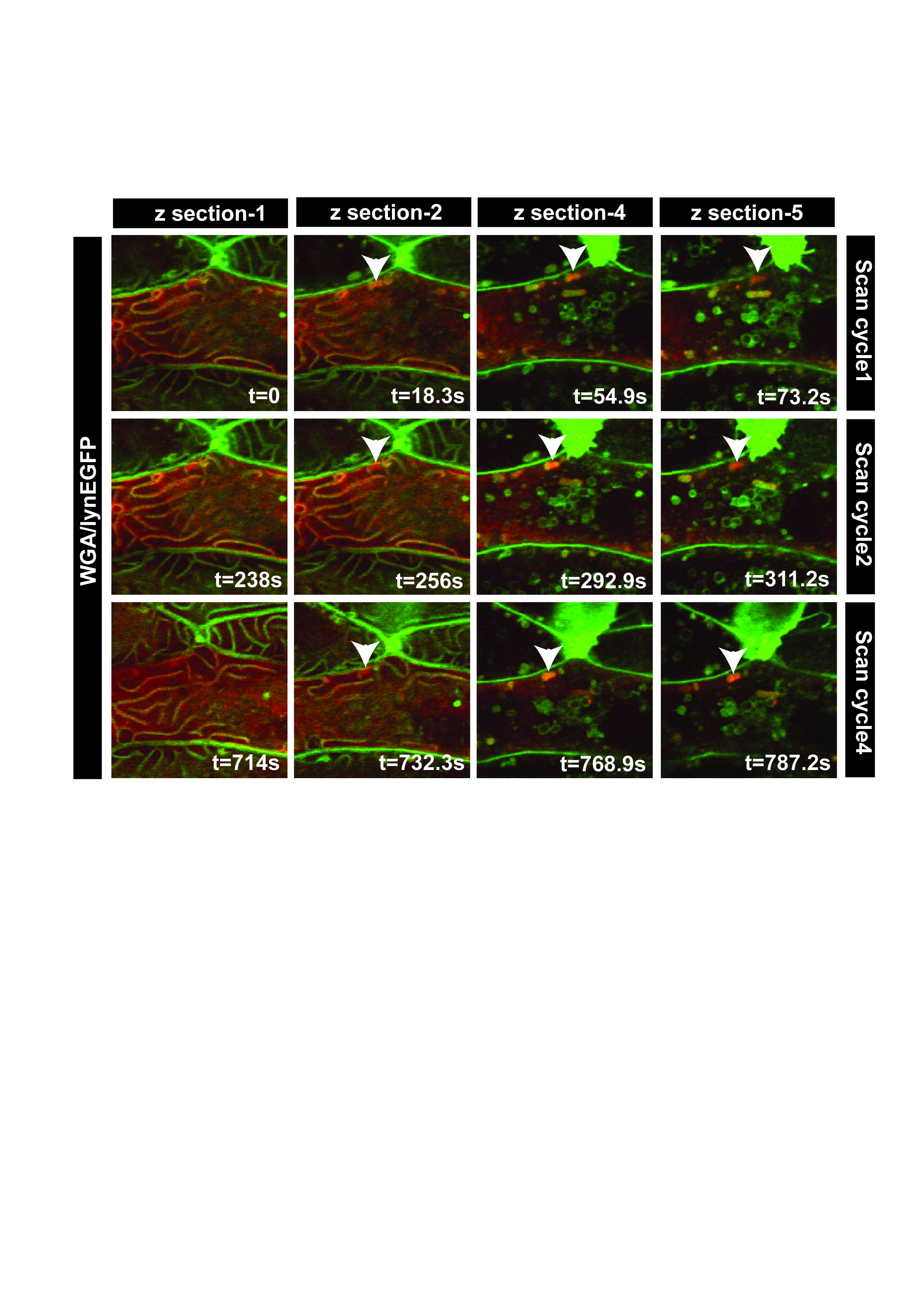

Supplement: Figure S4 — Live time-lapse analysis of WGA uptake in myoVb morphants in lynEGFP background across the indicated scan-cycles and z-sections. The arrowheads indicate formation and endocytosis of an apical vesicle. Note the disappearance of the vesicle in Z section −2 between 18.3 and 732.3 seconds. In section-5, this vesicle emerges as time progresses from 73.2 to 787.2 seconds. t = relative frame acquisition time. (JPG) [file pgen.1004614.s004.jpg]

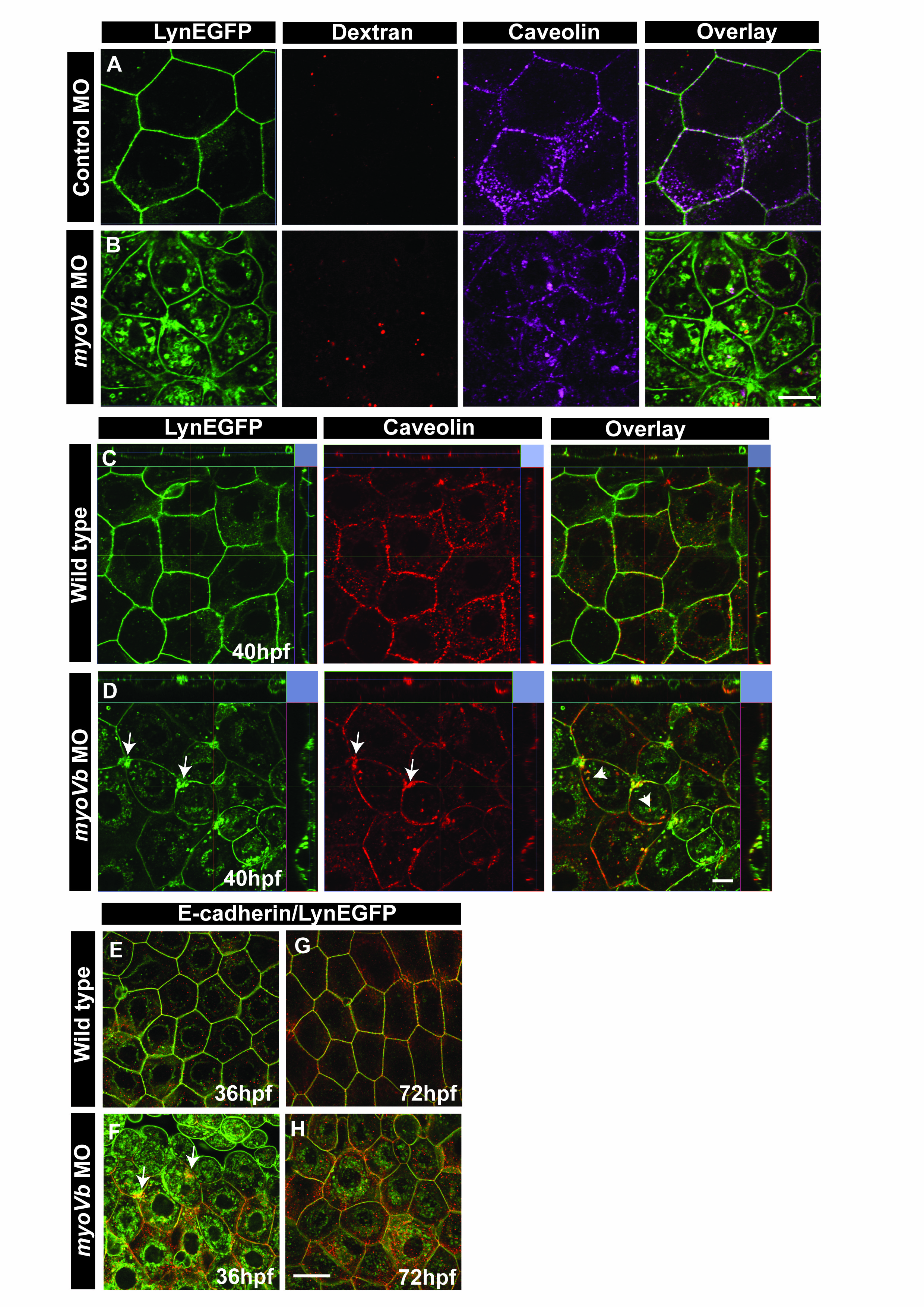

Supplement: Figure S5 — Simultaneous labelling using LynEGFP, Alexa 546 conjugated Dextran and anti caveolin antibody in control (A) and myoVb (B) morpholino injected embryos. The caveolin and Dextran label do not co-localise suggesting that Dextran enters in the cytoplasm only from the apical side of the peridermal cells. Immuno-localisation of Caveolin and LynEGFP in wild-type (C) and myoVb morphant (D) embryos at 40hpf. Arrows indicate spurts of endocytosis in the morphants whereas arrowheads indicate internalised caveolin vesicles. Overlay for E-cadherin (red) and LynEGFP (E–H) reveals that the endocytic spurts in the morphants (arrows in F) are labelled for E-cadherin at 48hpf and the frequency of the spurts decreases by 72hpf (H). Scale bars in B and D are equivalent to 10 µ whereas in H is equal to 20 µ. (JPG) [file pgen.1004614.s005.jpg]

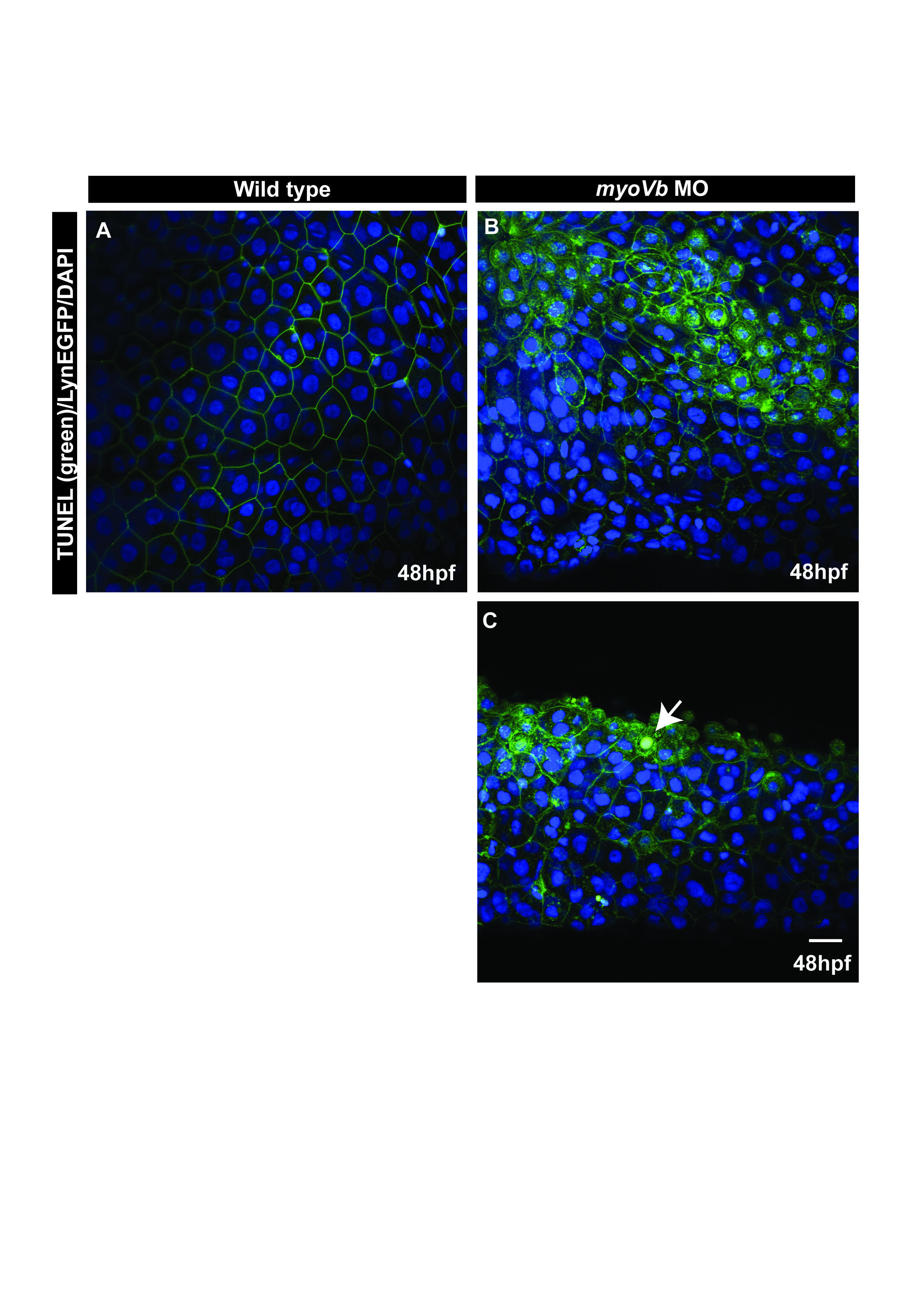

Supplement: Figure S6 — TUNEL (green, nuclear), LynEGFP (green, membrane label) and DAPI staining (blue) of wild type (A) and morphant embryos (B, C) at 36hpf. The arrow (C) indicates nuclear TUNEL staining in the myoVb morphant periderm. Scale bar corresponds to 20 µ. (JPG) [file pgen.1004614.s006.jpg]

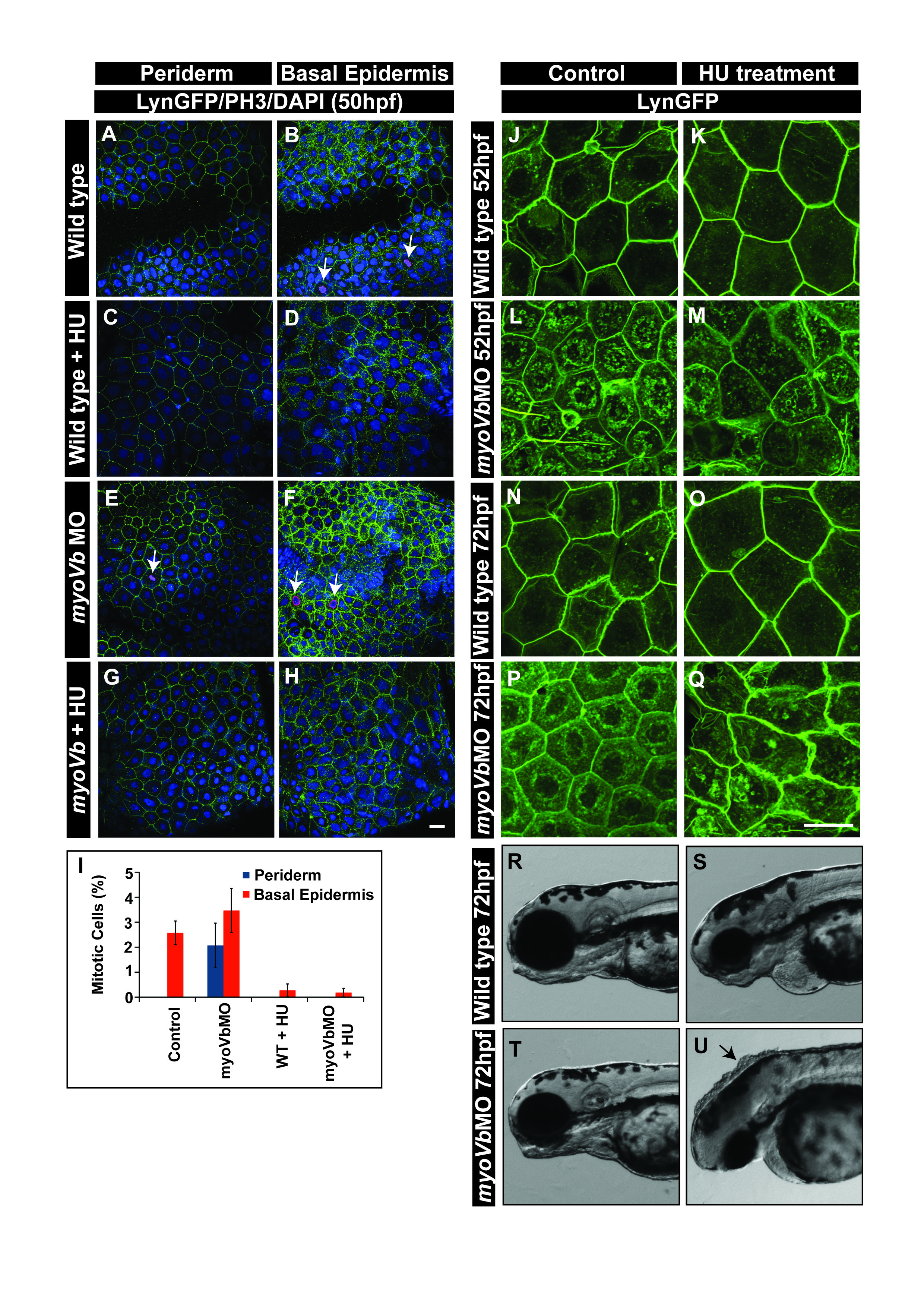

Supplement: Figure S7 — LynEGFP (green), phosphohistone-3 (red) and DAPI (blue) staining in wild type peridermal cell (A) and basal epidermal cells (B); HU treated periderm (C) and basal epidermis (D); myoVb morphant periderm (E) and basal epidermis (F); HU treated morphant periderm (G) and basal epidermis (H). Quantification of mitotic indices (I) under genetic condition and treatments mentioned along the X-axis. In myosin morphants peridermal cells exhibit increased mitosis. HU treatment inhibits proliferation in the basal epidermis of wild type and in the basal epidermis as well as periderm of myoVb morphants. LynEGFP staining in periderm of wild type control at 52 and 72 hpf (J, N), HU treated wild type (K,O), myoVb morphant (L,P) and HU treated myoVb morphant (M,Q). Note increased cell size upon HU treatment in wild type larvae (K,O) and irregular peridermal cell shapes in the HU treated myoVb morphants (Q). DIC images of wild type control (R), HU treated wild type (S), myoVb morphant (T) and HU treated myoVb morphant larvae (U) at 72hpf. While the peridermal cell rounding phenotype over the head recovers by 72hpf in myoVb morphants, it persists in HU treated morphants (arrow in U). Scale bars in H and Q correspond to 20 µ in A–H and J–Q, respectively. (JPG) [file pgen.1004614.s007.jpg]

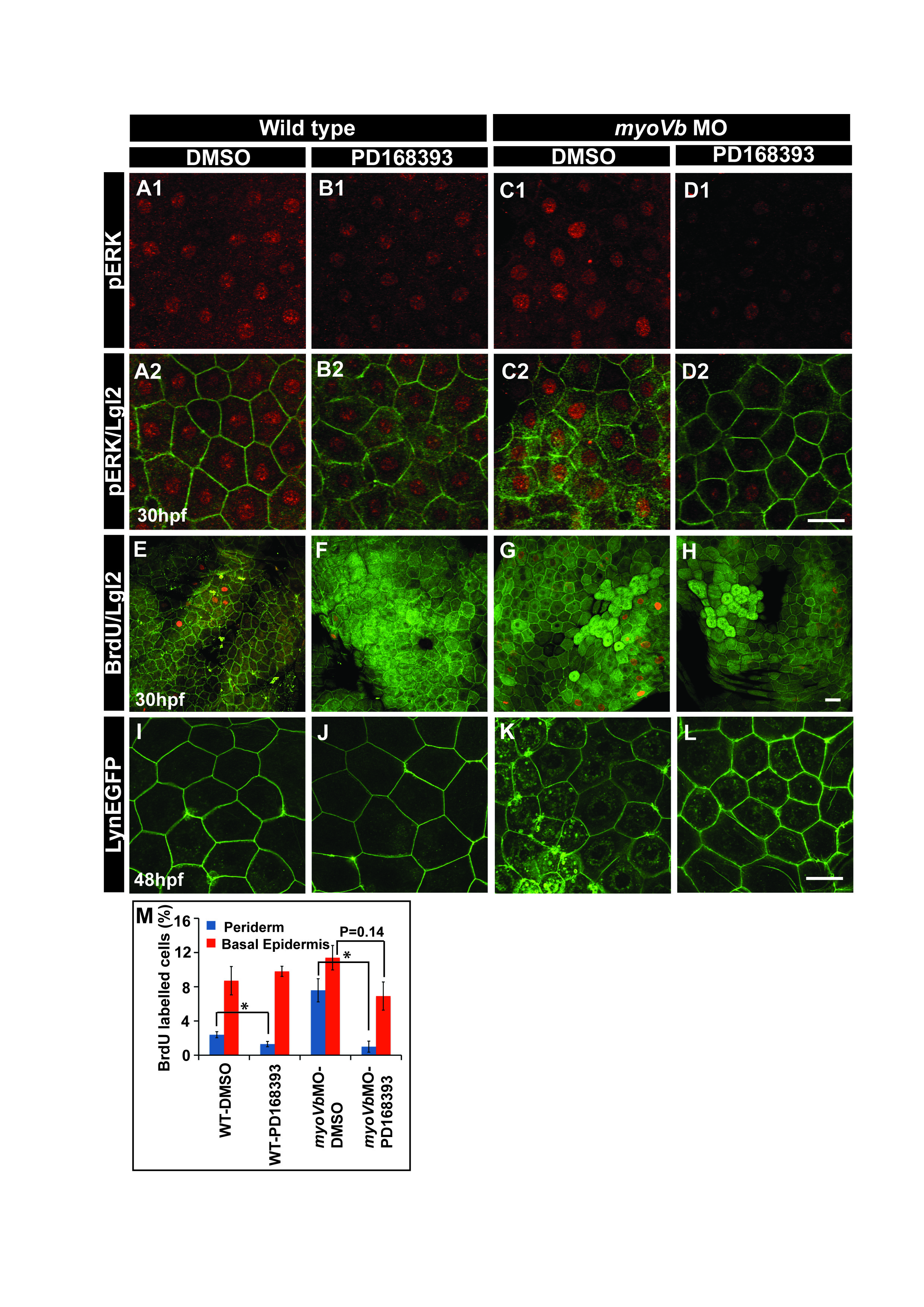

Supplement: Figure S8 — pERK (A1,B1,C1,D1) and pERK/LynEGFP overlays (A1,B2,C2,D2) in wild type (A1,A2,B1,B2) and myoVb morphants (C1,C2,D1,D2) treated with DMSO (A1,A2,C1,C2) and PD168393 (B1,B2,D1,D2) at 30hpf. Note the clear decrease in pERK in morphants treated with PD168393. Analysis of cell proliferation using BrdU (E,F,G,H) at 30hpf and cell size using lynEGFP line at 48hpf (I,J,K,L) in wild type (E,F,I,J) and myosin Vb morphant (G,H,K,L) treated with either DMSO (E,I,G,K) or PD168393 (F,J,H,L). In ‘H’ the few BrdU labelled nuclei of the basal epidermis are in focus at the periphery of the field. Quantification of cell proliferation in periderm and basal epidermis (M) in various genetic conditions and treatments shown across the X-axis. Scale bars in D2, L and H corresponds to 20 µ in A1–D2,I–L and in E–H, respectively. The square brackets and asterisk in (M) represent the comparisons by T-test and significant difference at (p≤0.05). (JPG) [file pgen.1004614.s008.jpg]

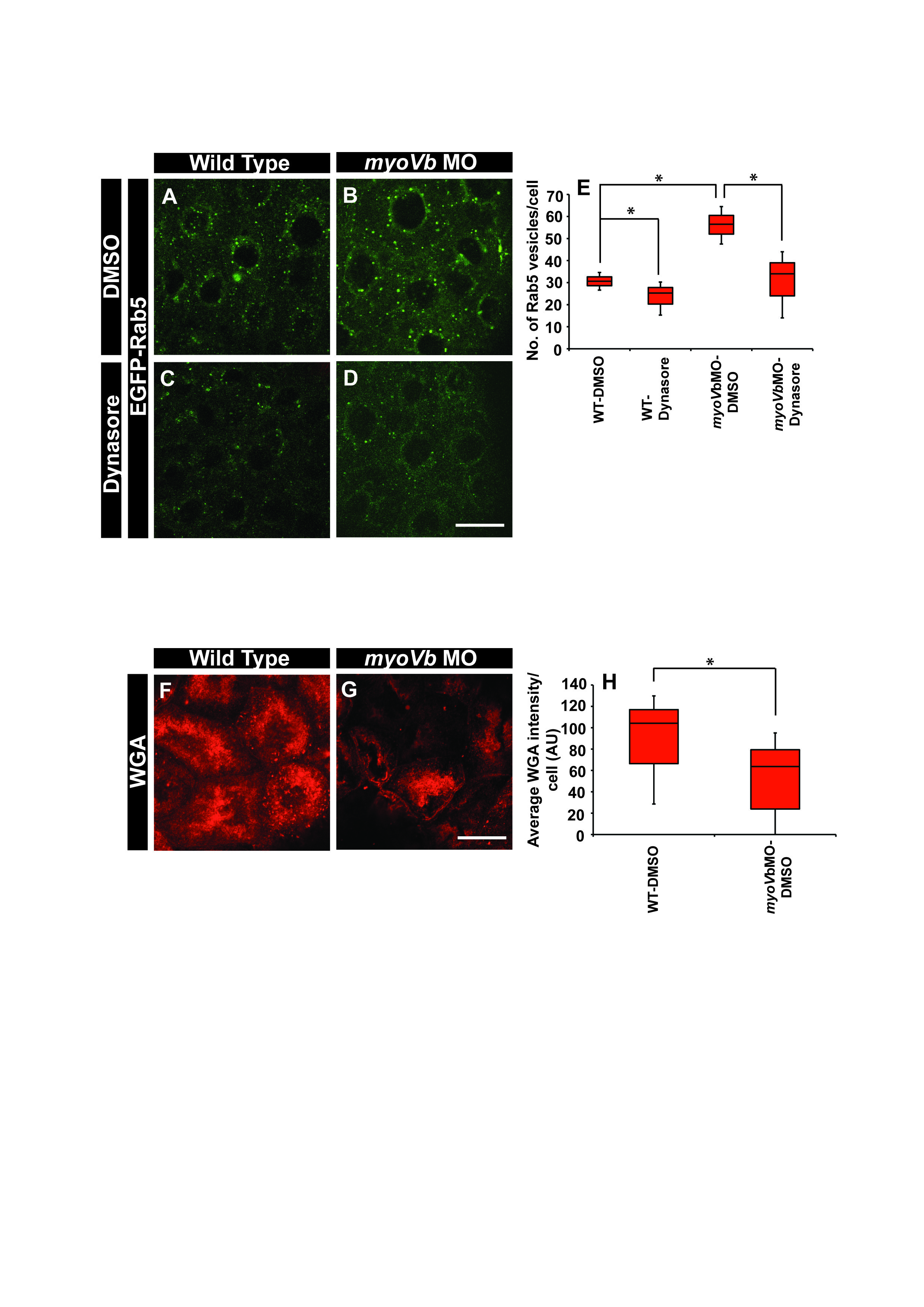

Supplement: Figure S9 — Live imaging of EGFP-Rab5 endosomes in wild type (A,C) and myoVb morphant embryos (B,D) treated with DMSO (A,B) and Dynasore (C,D) at 28hpf and their quantification (E). WGA labelling in wild type (F) and myosin Vb morphant embryos (G) at 28hpf. Quantification of the labelling intensities in wild type and morphant peridermal cells (H). Scale bars in D and G equals to 10 µ. The square brackets (in E, H) represent the comparisons and the asterisks indicate the significant difference (t test; P<0.01). (JPG) [file pgen.1004614.s009.jpg]
